# Lactate-Mediated Brain Acidosis Drives Epigenetic Dysregulation of TGFB2 and Associated Gene Networks in Schizophrenia and Bipolar Disorder

**DOI:** 10.3390/ijms27125456

**Published:** 2026-06-17

**Authors:** Hamid Mostafavi Abdolmaleky, Shabnam Nohesara, Reshma Subramonian, Kodhai Duraiarasan, Melissa Dorzin, Jin-Rong Zhou, Giuseppe Pettinato, Sam Thiagalingam

**Affiliations:** 1 Department of Medicine, Division of Gastroenterology, Beth Israel Deaconess Medical Center, Harvard Medical School, Boston, MA 02215, USA; sabdolma@bidmc.harvard.edu (H.M.A.); mdorzin@bidmc.harvard.edu (M.D.); 2 Stem Cells and Organoid Research Engineering (SCORE) Center, Beth Israel Deaconess Medical Center, Harvard Medical School, Botson, MA 02215, USA; 3 Department of Medicine (Biomedical Genetics), Boston University Chobanian & Avedisian School of Medicine, Boston, MA 02118, USA; snohesar@bu.edu (S.N.); kodhaid@bu.edu (K.D.); 4 T.H. Chan School of Medicine, UMass Chan Medical School, Worcester, MA 01655, USA; reshma.subramonian@umassmed.edu; 5 Department of Surgery, Beth Israel Deaconess Medical Center, Harvard Medical School, Boston, MA 02215, USA; jzhou10@bidmc.harvard.edu; 6 Department of Pathology & Laboratory Medicine, Boston University Chobanian & Avedisian School of Medicine, Boston, MA 02118, USA

**Keywords:** schizophrenia, bipolar disorder, DNA methylation, *TGFB2*, brain pH, lactic acid, iPSC, brain organoid, astrocyte, DNA hydroxymethylation

## Abstract

Gene expression analyses of postmortem brains have identified hundreds of dysregulated genes in schizophrenia (SCZ) and bipolar disorder (BD). Lactate accumulation and reduced brain pH are also consistently reported in these disorders. As increased TGFB expression has been implicated in major psychiatric diseases and lactic acid induces *TGFB2* upregulation in metabolic diseases, we hypothesized that lactate-induced brain acidosis may drive widespread gene dysregulation through TGFB2 activation. In our previous microarray studies, increased *TGFB2* expression was observed in postmortem brains of SCZ and BD patients, while pathway analyses suggested a key role for *TGFB2* in the dysregulation of other genes, particularly astrocytic genes. *TGFB2* itself also exhibited promoter DNA hypomethylation in postmortem brains of these patients. Here, while brain pH was lower in SCZ and BD patients, we investigated the effects of pH alteration on the expression and promoter DNA methylation of *TGFB2* and *TGFB2*-correlated genes in iPSC-derived neurons, astrocytes, and brain organoids (brainoids). Cultures were treated with lactic acid, HCl, bicarbonate, or NaOH to alter culture medium pH by ±0.4 units, and gene expression and promoter DNA methylation were evaluated by qPCR analyses. In our reanalysis of postmortem brain microarray data, nearly 80% of dysregulated genes, together with *TGFB2*, exhibited inverse correlations with brain pH. Lactic acid treatment induced increased expression and promoter DNA hypomethylation of *TGFB2* and several correlated genes in astrocytes and brainoids, whereas bicarbonate and NaOH treatments showed opposite effects. These findings suggest that lactate-mediated brain acidosis may contribute to *TGFB2* upregulation and widespread gene dysregulation implicated in SCZ and BD pathogenesis. Therapeutic interventions targeting lactic acid accumulation or *TGFB2* hyperexpression may mitigate disease-associated brain gene dysregulation.

## 1. Introduction

Major mental illnesses such as schizophrenia (SCZ) and bipolar disorder (BD) are chronic and debilitating neuropsychiatric disorders characterized by symptoms including delusions, hallucinations, and behavioral dysregulation, each affecting approximately 0.5–1% of the global population [1,2,3]. Affected individuals often experience lifelong impairment, with only partial symptomatic relief from antipsychotic medications or mood stabilizers. Consequently, many patients remain functionally impaired, imposing a substantial burden on families and society [4,5].

Although these disorders are highly heritable, the underlying molecular mechanisms contributing to their complex pathogenesis remain largely unknown [6,7,8]. Large-scale genetic studies have identified hundreds of genetic polymorphisms, each conferring modest effect sizes, that collectively contribute to the risk of SCZ, BD, and autism spectrum disorders [9,10,11]. In addition to genetic susceptibility, accumulating evidence over the last two decades indicates that metabolic dysfunction and alterations in the gut microbiome, both of which can influence the epigenome, are also implicated in the pathophysiology of these disorders [12,13,14,15]. Collectively, these findings suggest that the etiology and development of effective therapeutic strategies for neuropsychiatric disorders may be better understood through integrative frameworks that incorporate genetic, epigenetic, and environmental factors, particularly those influencing interconnected neural signaling pathways [16,17,18]. Metabolic abnormalities are more prevalent in SCZ and BD, including in drug-naïve patients and their first-degree relatives, suggesting that these disturbances are not solely medication-induced. Accumulating evidence indicates that such metabolic dysregulations, potentially driven by mitochondrial dysfunction and gut microbiota dysbiosis, may contribute to or arise from underlying epigenetic aberrations observed in these patients [16,19]. Epigenetic mechanisms represent critical regulatory interfaces that link cellular metabolic states and environmental exposures to the functional output of the genome, thereby enabling dynamic fine-tuning of gene expression in response to internal (e.g., metabolic) and external perturbations [17,18].

A well-recognized consequence of mitochondrial dysfunction and metabolic dysregulation is a shift in intracellular and tissue pH toward acidification [20,21]. In the context of neuropsychiatric disorders, multiple studies have reported reduced brain pH in SCZ and BD, frequently accompanied by elevated lactate levels [22]. This inverse relationship between pH and lactate has been further substantiated by meta-analyses encompassing more than ten independent studies [23,24,25]. Notably, brain pH has been shown to remain relatively stable during the early postmortem interval (0–48 h), and may even increase during freezer storage [26,27]. Therefore, the observed association between reduced brain pH and psychiatric disorders is unlikely to be attributable to postmortem preservation conditions.

Elevated brain lactate levels and reduced brain pH have also been reported across several neuropsychiatric and neurodegenerative conditions, including Alzheimer’s disease and autism spectrum disorders, as well as in drug-naïve animal models of psychiatric disease [24]. Supporting the broader relevance of this phenomenon, a meta-analysis of 281 human transcriptomic datasets spanning 11 brain disorders demonstrated that gene expression signatures associated with reduced brain pH are significantly overrepresented across multiple conditions, including SCZ, BD, autism spectrum disorders, Alzheimer’s disease, Parkinson’s disease, and Huntington’s disease [28].

**Figure 1:**
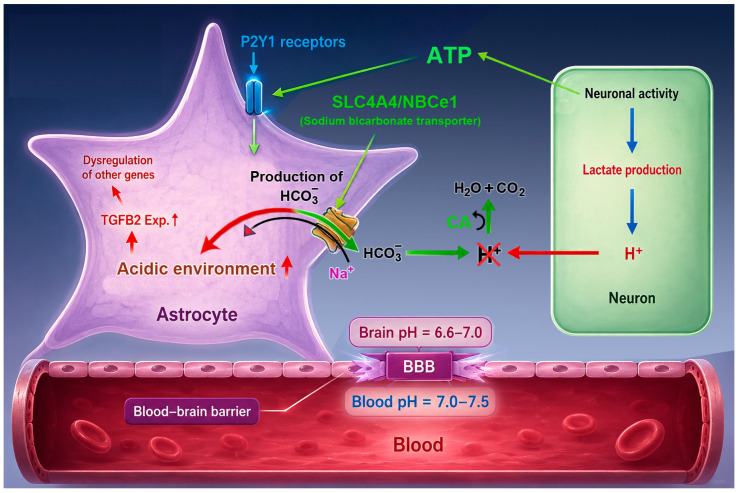
Proposed model linking neuronal activity, lactate metabolism, and brain pH regulation. Neuronal activity is associated with increased lactate production, contributing to local acidification and reduced brain pH. ATP (adenosine triphosphate) released during neuronal activity activates astrocytic P2Y1 (Purinergic Receptor P2Y1) receptors, triggering calcium signaling and astrocyte-mediated pH regulation. Astrocytes help regulate extracellular pH by producing bicarbonate and transporting it via SLC4A4 to the intercellular space, where it buffers excess H^+^. This buffering process contributes to a relatively more acidic intracellular pH in astrocytes compared to other brain cell types. Notably, brain pH is lower than blood pH (6.6–7.0 vs. 7.0–7.5). In peripheral adipose tissue, lactate has been shown to induce *TGFB2* expression [30]. Consistently, our previous findings suggest that lactate-associated reductions in brain pH may also be linked to increased *TGFB2* expression in postmortem brains of patients with SCZ [31].

Notably, consistent with prior experimental observations, astrocytes were found to express the highest proportion of “acidity-associated” genes and to exhibit a relatively lower intracellular pH compared to neurons. Functionally, this aligns with their central role in pH homeostasis. As shown in Figure 1 [22], while neuronal activity increases H^+^ production and promotes local acidification, contributing to lower brain interstitial pH relative to blood (approximately 6.6–7.0 vs. 7.0–7.5), astrocytes counterbalance these changes through bicarbonate production and export, mediated in part by electrogenic sodium bicarbonate cotransporter 1 (NBCe1/SLC4A4) [29]. This buffering function, however, results in a comparatively more acidic intracellular environment within astrocytes.

As a substantial body of experimental evidence indicates that increased brain lactate accompanied by decreased brain pH is a common feature of SCZ and other major neuropsychiatric and neurodegenerative disorders [24,25], a landmark mechanistic study in metabolic disease demonstrated that lactate can upregulate TGFB2 expression, whereas pharmacological inhibition of lactate signaling attenuates this effect [30]. Consistent with these findings, our prior gene expression microarray analysis of postmortem brain tissue from patients with SCZ and BD compared with well-matched controls (*N* = 10 per group) identified hundreds of differentially expressed genes. Notably, the most significantly dysregulated gene sets were enriched for astrocyte differentiation and function, and transforming growth factor beta (TGFβ) signaling emerged as a key upregulated pathway [31]. These observations are consistent with earlier meta-analytic evidence suggesting that TGFβ acts as a state-related biomarker in SCZ, as its expression is elevated in first-episode psychosis and acute relapse, but is reduced following antipsychotic treatment [32].

Given the close relationship between brain lactate levels and brain pH, it is reasonable to propose that lactate-induced acidification of the brain may contribute to or orchestrate gene expression alterations observed in the brains of patients with major mental disorders. This mechanism may therefore represent a potential therapeutic target. However, ethical and practical constraints preclude direct mechanistic experimentation in the living human brain, limiting opportunities to test such hypotheses and develop novel interventions in situ. In addition, substantial differences in physiology, genetics, epigenetic regulation, and developmental trajectories between human and animal brains continue to pose major challenges for the translational validity of preclinical drug studies. Nevertheless, human induced pluripotent stem cells (hiPSCs), with their capacity for self-renewal and differentiation into diverse neural cell types and brain organoids (brainoids), provide a powerful platform to partially overcome these limitations and enable human-relevant modeling of disease-associated molecular and metabolic processes. Here, based on the hypothesis that lactate- and pH-mediated gene dysregulation contributes to the pathogenesis of SCZ or BD and other psychotic disorders, we reanalyzed our previously published whole-transcriptome data from postmortem brain samples of patients with SCZ and BD in relation to brain pH compared with matched control subjects [31].

In addition, we investigated the effects of lactic acid, HCl, bicarbonate, and NaOH on pH-associated gene expression and DNA methylation, including DNA hydroxymethylation, in human induced pluripotent stem cell (hiPSC)-derived neural stem cells (NSCs), neurons, astrocytes, and brainoids. This in vitro model system was designed to recapitulate brain molecular features relevant to neuropsychiatric disease. Together, these approaches may provide a framework for identifying and testing novel therapeutic strategies aimed at modulating brain pH-related molecular pathways in SCZ, BD, and potentially other psychotic disorders.

## 2. Results

Developing effective treatments for SCZ and/or BD remains challenging, because the lack of suitable experimental models and the extreme complexity of the neural circuits have led to an incomplete understanding of the underlying pathological processes. However, one of the valuable observations from the study of postmortem brains is the fact that lactate causes an increase in *TGFB2* expression as a key regulator of SCZ or BD pathogenesis and anti-lactate drugs inhibit this effect [30]. Therefore, we extended the analysis of our gene expression profiling data of postmortem brain samples from controls and patients with SCZ and BD to include the pH of the brain to uncover significant correlations among gene expression patterns and test these correlations in renewable model systems for these diseases.

### 2.1. Brain pH Is Lower in Postmortem Brains of SCZ and BD Patients, Which Exhibit Increased Expression of TGFB2 and TGFB2-Correlated Genes

We analyzed brain pH in a total of 105 postmortem frontal lobe samples from patients with SCZ and BD compared with matched control subjects (*N* = 35 per group), obtained from the Stanley Medical Research Foundation, in which pH was measured during tissue processing. The observed pH range was 5.76–7.03 overall, 6.00–7.03 in controls, 5.90–6.93 in SCZ, and 5.76–6.97 in BD. Brain pH was significantly lower in both SCZ and BD patients compared with controls (6.48 and 6.43 vs. 6.61, respectively; *p* = 0.03 and *p* = 0.01; two-tailed *t*-test). In psychotic BD patients, pH was also significantly lower than non-psychotic BD patients (6.35 vs. 6.57, *p* = 0.04, two-tailed *t*-test).

In our previous gene expression microarray analysis of postmortem brain tissue from SCZ and BD patients versus well-matched controls (*N* = 10 per group) [31], using nominal *p*-values, we found that although hundreds of genes were differentially expressed, TGF-β signaling emerged as a significantly upregulated pathway in both disorders. Additionally, genes involved in astrocyte-related functions represented the major affected pathways in gene set enrichment analysis (GSEA) (see Abdolmaleky et al., 2019 [31] for details). Consistent with the microarray findings, confirmatory qRT-PCR analysis in the full cohort (*N* = 35 per group vs. controls) demonstrated significantly increased expression of *TGFB2* in postmortem brain tissue from patients with SCZ and BD [31].

**Figure 2:**
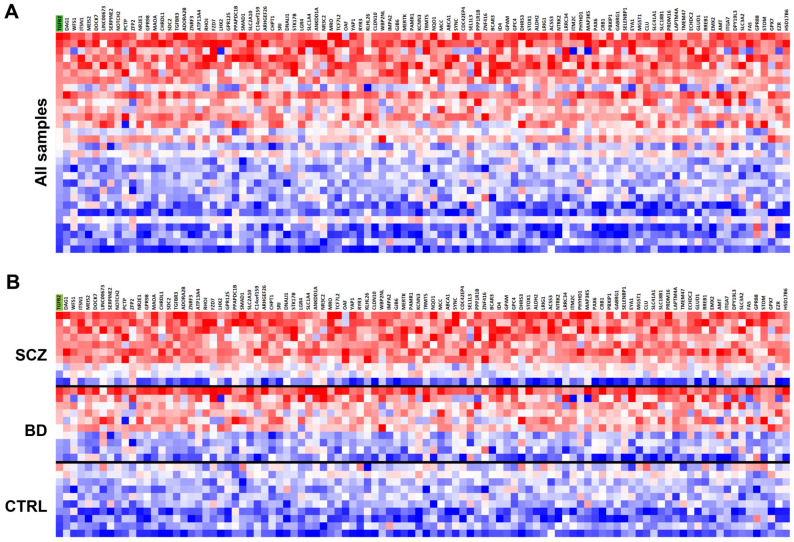
Key dysregulated genes in SCZ and BD are associated with TGFB signaling. (**A**) Overall, increased *TGFB2* expression is positively correlated with the expression of more than 300 genes. (**B**) This correlation pattern is also observed in schizophrenia (SCZ), bipolar disorder (BD), and control subjects. Here, the correlation of 100 representative genes is shown (for the complete gene set, see Supplementary File S1, Sheets S1 and S2). Expression levels are color-coded (blue/white/red = below/at/above average expression).

In subsequent complementary analyses, we found that more than 75% of genes with increased expression in SCZ and BD exhibited positive correlations with *TGFB2* expression (Figure 2) and negative correlations with brain pH (r > ±0.4, *p* < 0.03). Consistently, TGFB2 expression itself showed a significant inverse correlation with brain pH (r = –0.54, *p* = 0.002).

**Figure 3:**
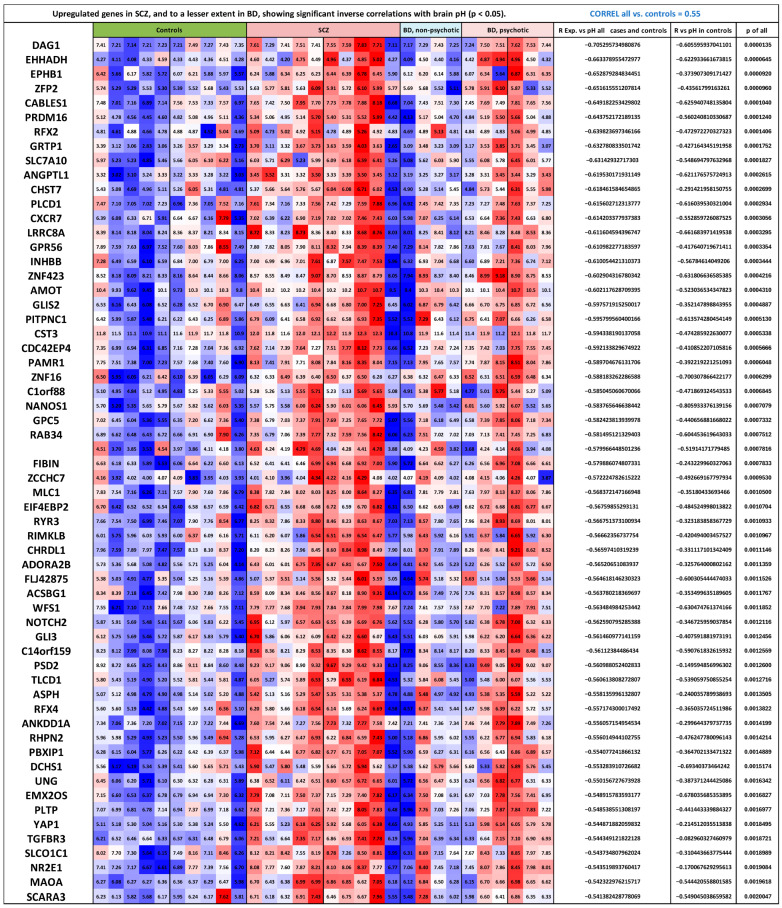
Upregulated genes inversely correlated with brain pH in SCZ. The first 60 genes (of 315 total) showing highly significant inverse correlation between expression and brain pH (*p* < 0.002) in SCZ are shown. A similar expression pattern is observed in BD, particularly in psychotic BD. The complete list of all 315 genes is provided in Supplementary File S1, Sheet S3. Expression levels are color-coded (blue/white/red = below/at/above average expression).

**Figure 4:**
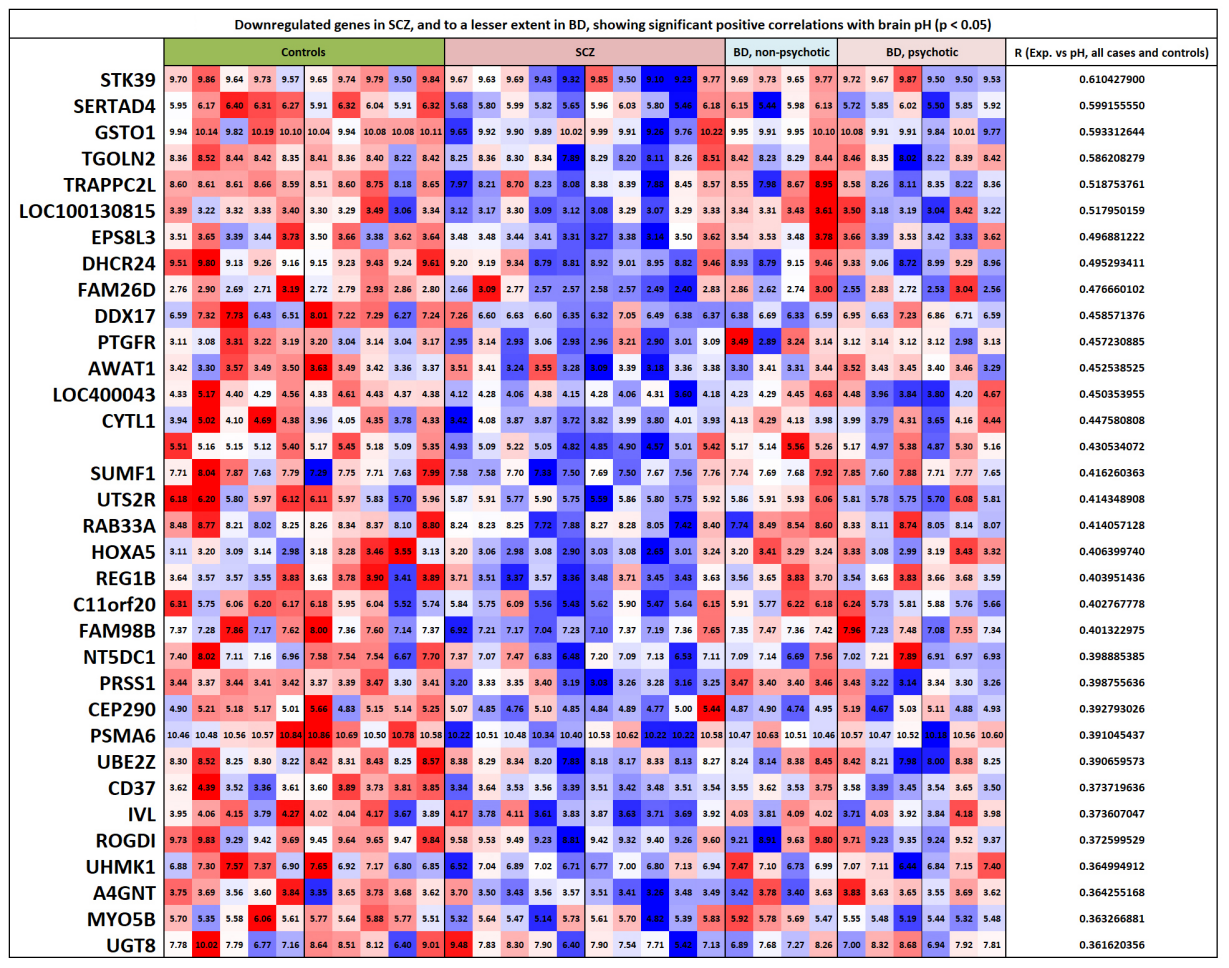
Downregulated genes positively correlated with brain pH in SCZ. Among 284 genes whose expression showed a significant positive correlation with brain pH (*p* < 0.05), only 34 genes (12%) were significantly downregulated in SCZ. A similar trend is observed in BD, particularly in psychotic BD; however, many expression changes in BD did not reach statistical significance compared with control subjects. Expression levels are color-coded (blue/white/red = below/at/above average expression).

Analysis of the relationship between brain pH and gene dysregulation revealed that at the nominal significance threshold (*p* < 0.05), approximately 80% of dysregulated genes in SCZ and BD showed increased expression, the majority of which were also correlated with TGFB2 expression. Furthermore, 79% of all dysregulated genes exhibited a significant correlation with brain pH (either positive or negative). In SCZ, 95% of genes, and in BD, 97% of genes with >20% expression changes showed increased expression accompanied by an inverse correlation with pH. Notably, among 315 genes whose expression was inversely correlated with pH at a nominal significance level (r < –0.361, *p* < 0.05), nearly all demonstrated significant upregulation in SCZ and to a large extent in BD (Figure 3 and Supplementary File S1, Sheet S3). In contrast, among 284 genes showing a positive correlation with pH (r > 0.361, *p* < 0.05), 34 genes (12%) were downregulated in SCZ (Figure 4), whereas none showed upregulation in either SCZ or psychotic BD. Because these analyses involved hundreds of simultaneous gene-level correlations, we primarily applied the nominal *p* < 0.05 threshold in an exploratory, hypothesis-generating context rather than as definitive evidence of statistical significance. We also applied Benjamini–Hochberg false-discovery rate (FDR) correction within the set of 315 genes identified as inversely correlated with pH. Using an FDR threshold of q < 0.25, 190 of 315 genes remained significant and were prioritized for downstream validation analyses. Nevertheless, under a more lenient threshold (q < 0.3), nearly all 315 genes met significance criteria (Supplementary File S1, Sheet S3), further supporting the robustness of the observed association signal in this exploratory dataset. The principal biological interpretation, however, is based on convergence across independent layers of evidence, including directionally consistent postmortem pH correlations, TGFB2-associated gene networks, and experimental validation of selected candidate genes in astrocytes and brainoids.

It is worth noting that the r values of the 315 genes exhibiting strong inverse correlations with pH were highly significantly correlated with the corresponding r values observed in the control group (r = 0.55, *p* = 2.66 × 10^−26^). However, this correlation was negligible for the 284 genes exhibiting direct correlations with pH (r = 0.085), as well as for genes whose expression showed strong positive correlations with pH (r > 0.463, *p* < 0.01), with a correlation coefficient of only r = 0.14 (*p* = 0.36). Collectively, these findings together with the data presented in Figure 2, suggest that reduced brain pH is strongly associated with widespread gene expression upregulation in both control subjects and in individuals with SCZ and BD. However, this effect is more pronounced in the disease state, where brain pH is lower.

On Pearson correlation analysis, expression changes of only 18% of SCZ-associated genes (19.3% of 315 upregulated genes and 17.6% of 34 downregulated genes) showed significant positive or negative correlations with the extent of antipsychotic drug use (r = ±0.44, *p* < 0.05). Antipsychotic drug use was defined as cumulative antipsychotic intake expressed in fluphenazine-equivalent milligrams up to the time of death, as described in Materials and Methods. In BD, only 4.3% of differentially expressed genes exhibited a significant correlation with antipsychotic exposure at the same threshold. More specifically, among 295 genes with highly significant increased expression in SCZ (*p* < 0.01), 38 genes (13%) showed a positive correlation with antipsychotic exposure. Conversely, among 103 genes with highly significant decreased expression in SCZ (*p* < 0.01), only 11 genes (10.7%) showed a significant inverse correlation with antipsychotic drug use, suggesting that their expression changes may be partially attributable to medication effects. Notably, 166 of these 295 upregulated genes in SCZ (56%) exhibited a significant inverse correlation with brain pH, whereas none showed a positive correlation with pH.

As a longer postmortem interval (PMI) and refrigeration interval have been reported to have no effect or even to increase (rather than decrease) brain pH [26,27], we further examined the relationship between brain pH and refrigeration interval in our dataset. In the combined sample, the mean refrigeration interval in SCZ was significantly longer than in controls (6.0 h vs. 3.6 h; *p* = 0.008, two-tailed *t*-test). Notably, a significant positive correlation was observed between refrigeration interval and brain pH in SCZ (*n* = 35; r = 0.38, *p* = 0.024). Postmortem interval (PMI) was also approximately 2 h longer in SCZ compared with controls; however, this difference did not reach statistical significance. However, in SCZ, a significant positive correlation was observed between PMI and brain pH (r = 0.36, *p* = 0.037; two-tailed test). In BD, the refrigeration interval was also significantly longer than in controls (10.1 h vs. 3.6 h; *p* = 0.001), as was PMI (38 h vs. 29 h; *p* = 0.027).

Taken together, these findings suggest that the impact of reduced brain pH on gene expression alterations in SCZ and BD may be underestimated due to longer postmortem and refrigeration intervals in these patient groups. Indeed, extended refrigeration and postmortem intervals may contribute to an increase in measured brain pH, potentially partially compensating for or obscuring the lower in vivo brain pH present at the time of death in SCZ and BD subjects.

Following these findings, we conducted complementary analyses of the microarray dataset focusing on additional genes, particularly astrocyte-related genes associated with *TGFB2*, in relation to brain pH. For example, *TGFBR3* expression showed a strong positive correlation with *TGFB2* expression overall (r = 0.67, *p* = 5 × 10^−5^), as well as within SCZ (r = 0.83, *p* = 0.003) and BD (r = 0.73, *p* = 0.016). In addition, *TGFBR3* expression was significantly elevated in SCZ and psychotic BD (56% and 35%, respectively) and exhibited a significant inverse correlation with brain pH (r = −0.54, *p* = 0.002). Similarly, *SMAD1*, a downstream mediator of TGFB signaling, was significantly upregulated in SCZ (30%, *p* = 0.00095) and BD (23%, *p* = 0.016). *SMAD1* expression also showed a strong positive correlation with TGFB2 expression (r = 0.71, *p* = 1 × 10^−5^) and a significant inverse correlation with brain pH (r = −0.43, *p* = 0.017). The same pattern was observed for *BMP7*, a member of the TGFB signaling superfamily, which showed increased expression in both SCZ (26%) and BD (20%). *BMP7* expression was inversely correlated with brain pH (r = −0.38, *p* = 0.038) and positively correlated with *TGFB2* expression (r = 0.49, *p* = 0.006). Similarly, *BMPR1B* exhibited increased expression in SCZ (48%, *p* = 0.01) and BD (59%, *p* = 0.005), along with a significant inverse correlation with brain pH (r = −0.48, *p* = 0.007) and a strong positive correlation with *TGFB2* expression (r = 0.83, *p* = 1 × 10^−7^). *BMPR1A* also showed increased expression in SCZ (26%, *p* = 0.02) and BD (32%, *p* = 0.03), as well as a positive correlation with *TGFB2* expression (r = 0.49, *p* = 0.006); however, it did not exhibit a significant inverse correlation with brain pH (r = −0.16).

*SLC1A2*, an astrocytic gene previously reported to be inversely correlated with brain pH in SCZ [33], showed more than a 200% increase in expression in both SCZ and psychotic BD (*p* = 0.0076 and *p* = 0.05, respectively) in our microarray analysis. In addition, *SLC1A2* expression was positively correlated with *TGFB2* expression (r = 0.70, *p* = 0.0001) and exhibited a trend toward an inverse correlation with brain pH (r = −0.35, *p* = 0.057; two-tailed test). Similarly, *SLC1A3*, another astrocytic gene [29] and previously shown to be upregulated by reduced brain pH [34], exhibited increased expression in both SCZ and BD (55%, *p* = 0.018 and *p* = 0.023, respectively). *SLC1A3* expression also showed a strong positive correlation with *TGFB2* expression overall (r = 0.85, *p* = 1 × 10^−7^) and a significant inverse correlation with brain pH (r = −0.45, *p* = 0.01).

*SLC4A4*, another key astrocytic regulator of brain pH that mediates bicarbonate transport from astrocytes to the extracellular space (thereby contributing to extracellular pH homeostasis) [29], exhibited significantly increased expression in both SCZ (38%, *p* = 0.024) and psychotic BD (48%, *p* = 0.017) compared with controls, suggestive of a potential reactive response. *SLC4A4* expression showed a strong positive correlation with *TGFB2* expression overall (r = 0.78, *p* = 4 × 10^−7^), as well as within SCZ (r = 0.87, *p* = 0.008) and BD (r = 0.70, *p* = 0.024), and a trend-level correlation in controls (r = 0.59, *p* = 0.07). In addition, *SLC4A4* expression exhibited a significant inverse correlation with brain pH both in the combined dataset (r = −0.42, *p* = 0.02) and in SCZ and BD (r = −0.50, *p* = 0.024).

The expression of *SLC16A1* (*MCT1*), a monocarboxylate transporter that transports lactate (primarily inward and likely bidirectional) and other monocarboxylates, showed no significant changes and no correlation with brain pH in any group. In contrast, *SLC16A3* (*MCT4*), an outward lactate transporter, exhibited a significant positive correlation with *TGFB2* expression in controls (r = 0.79, *p* = 0.006), but not in SCZ or BD (r = 0.20, n.s.). *SLC16A3* also showed a trend toward an inverse correlation with brain pH in controls (r = −0.49, *p* = 0.15) and a significant inverse correlation in SCZ (r = −0.65, *p* = 0.04), but not in BD (r = +0.49, *p* = 0.15). Notably, *SLC16A3* expression showed a significant correlation with antipsychotic drug use (r = 0.52, *p* = 0.02), suggesting that these medications may modulate lactate transport-related responses associated with altered brain pH in disease states.

Lactate is also known to regulate the expression of multiple genes, including *HIF1A* [35]. Consistently, *HIF1A* expression was increased in postmortem brains of patients with SCZ (*p* = 0.02) and showed a strong positive correlation with *TGFB2* expression overall (r = 0.81, *p* = 1 × 10^−7^). Finally, TNFA demonstrated a significant inverse correlation with brain pH both in the overall dataset (r = −0.49, *p* = 0.006) and in control subjects specifically (r = −0.65, *p* = 0.04). *TNFA* did not show a strong correlation with *TGFB2* expression (r = 0.22, n.s.), and its expression was not significantly altered in SCZ or BD. However, *TNFA* expression exhibited a positive correlation with antipsychotic drug use (r = 0.59, *p* = 0.006).

### 2.2. Carbonic Anhydrase (CA) Gene Expression Status in Postmortem Brains

With respect to genes involved in pH regulation, our expression microarray analysis of postmortem brain samples showed that *CA1* (a cytoplasmic enzyme predominantly expressed in erythrocytes and present at very low levels in brain tissue) exhibited a trend toward an inverse correlation with brain pH in control subjects (r = −0.47, *p* = 0.17), but showed no significant expression changes in SCZ or BD. However, *CA1* expression was positively correlated with antipsychotic drug use in SCZ and BD (r = 0.55, *p* = 0.012), with a stronger association observed in SCZ (r = 0.67, *p* = 0.033). These findings suggest that potential pH-related expression patterns of *CA1* in the disease state may be attenuated or obscured by antipsychotic treatment.

*CA2* (which is primarily expressed in glial cells) unexpectedly showed a trend toward a positive correlation with brain pH in control subjects (r = 0.56, *p* = 0.10), with a similar, albeit weaker trend in SCZ (r = 0.35), but not in BD (r = −0.14). Antipsychotic drug use did not significantly affect CA2 expression. We observed a significant reduction in the expression of *CA3* (carbonic anhydrase 3, predominantly expressed in the hippocampus) in SCZ (−18%, *p* = 0.017) and a trend toward reduction in BD (−11%, *p* = 0.10). Similarly, *CA5A* (a mitochondrial isoform) was significantly decreased in both SCZ (−13%, *p* = 0.03) and BD (−21%, *p* = 0.003). Notably, in control subjects, decreasing brain pH was associated with a trend toward increased expression of *CA3* and *CA5A* (r = −0.54 and r = −0.55, respectively; *p* = 0.11), whereas no such relationship was observed in SCZ or BD (r = −0.07 and r = 0.04, respectively). In control subjects, a trend toward an inverse correlation between brain pH and *CA9* expression was also observed (r = −0.59, *p* = 0.08). Although *CA9* expression was not significantly altered in SCZ or BD, it showed a trend toward a positive correlation with antipsychotic drug use overall (r = 0.43, *p* = 0.06), with a stronger and statistically significant association in SCZ (r = 0.75, *p* = 0.012).

*CA12* expression, predominantly localized to the choroid plexus, exhibited a strong inverse correlation with brain pH in control subjects (r = −0.80, *p* = 0.005), but not in SCZ or BD (combined r = 0.17), where no significant expression changes were observed. Similarly, *CA13* expression showed a trend toward an inverse correlation with brain pH both in the overall dataset (r = −0.35, *p* = 0.06) and in control subjects (r = −0.49, *p* = 0.16). Antipsychotic drug use was associated with increased *CA13* expression in SCZ (r = 0.57, *p* = 0.08), but not in BD (r = 0.39, n.s.).

These data suggest that as some key genes involved in pH regulation are dysregulated in SCZ and BD, antipsychotics may provide limited benefits. Nevertheless, the expression of *CA1* and *CA9*, which increased by antipsychotic drugs in SCZ, was found to be almost 16 times less than *CA2* (log_2_ values of 5 vs. 9.5), presenting much limited beneficial effects. On the other hand, the expression of *CA14*, with five times less expression compared to *CA2*, was reduced by antipsychotic drugs in SCZ (r = −0.89, *p* = 0.0005), but showed a trend for an increase in BD (r = 0.52, *p* = 0.12). Overall, since brain pH is reduced in SCZ and BD, it is expected that the expression of CA genes is increased to produce more bicarbonate ions (HCO_3_^−^) to stabilize brain pH in the disease states, which in contrast to control subjects was not observed in SCZ and BD.

### 2.3. Lactate Dehydrogenase (LDH) Gene Expression in Relation to Postmortem Brain pH

LDH enzymes catalyze the reversible conversion between lactate and pyruvate. They are composed of M and H subunits encoded by the *LDHA* and *LDHB* genes. Different combinations of these subunits form LDH isoenzymes (LDH1–LDH5), where the M subunit preferentially promotes pyruvate-to-lactate conversion, whereas the H subunit favors lactate-to-pyruvate conversion. None of these subunits showed significant expression changes in SCZ or BD or significant correlations with brain pH or antipsychotic drug use. However, *LDHD* showed a trend toward increased expression in SCZ (13%, *p* = 0.045) and exhibited a significant inverse correlation with brain pH overall (r = −0.46, *p* = 0.01), with a similar trend in controls (r = −0.46, *p* = 0.18). LDHD is localized to mitochondria and serves a metabolic housekeeping function by preventing accumulation of D-lactate, which has limited metabolic and functional relevance in humans, but can be neurotoxic at elevated levels [36]. Thus, increased *LDHD* expression in SCZ may represent a compensatory response to elevated D-lactate levels. *LDHD* expression also showed a significant positive correlation with *TGFB2* expression overall (r = 0.45, *p* = 0.01), as well as in control subjects (r = 0.67, *p* = 0.03), but no correlation with antipsychotic drug use (r = 0.16).

### 2.4. pH Effects on the Expression of Epigenetic Regulatory Genes in Postmortem Brain Samples

While lactic acid itself may induce epigenetic modifications through histone lactylation, we focused on gene expression changes involved in epigenetic regulation. On microarray analysis, we observed an inverse correlation between brain pH and expression of *IDH1* and *IDH2* (r = −0.32, *p* = 0.08 and r = −0.42, *p* = 0.02, respectively), encoding enzymes that potentiate the activity of TET family of DNA demethylation enzymes [37]. Furthermore, *IDH1* and *IDH2* expression levels were significantly increased in schizophrenia (SCZ) (21%, *p* = 0.037 and 17%, *p* = 0.01, respectively). Notably, *TGFB2* expression showed strong positive correlations with both *IDH1* and *IDH2* (r = 0.68 and r = 0.74, respectively; *p* < 0.00002). Consistent with these findings, the *TGFB2* promoter has been reported to exhibit DNA hypomethylation in the same postmortem brain samples from SCZ and BD patients [31].

*SIRT1*, which showed increased expression in psychotic BD and a trend-level increase in SCZ (34% and 20%, *p* = 0.01 and 0.057, respectively), exhibited a trend toward an inverse correlation with brain pH in BD (r = −0.45, *p* = 0.19), but not in SCZ (r = −0.1). In contrast, in control subjects *SIRT1* showed a trend toward a positive correlation with pH (r = 0.51, *p* = 0.13). Notably, SIRT1 expression was positively correlated with *TGFB2* expression both across groups (r = 0.52, *p* = 0.005) and in the combined SCZ and BD cohort (r = 0.50, *p* = 0.025), while antipsychotic drug use had no detectable effect on *SIRT1* expression (r = −0.004).

*SIRT2*, a cytoplasmic sirtuin, showed a non-significant increase in expression in both SCZ and BD (17% in each group). In contrast, antipsychotic drug use was associated with decreased *SIRT2* expression (r = 0.46, *p* = 0.04). The mitochondrial sirtuins, *SIRT3* and *SIRT4*, showed no significant expression changes in SCZ or BD. However, *SIRT3* expression was positively correlated with antipsychotic drug exposure (r = 0.45, *p* = 0.04).

The expression levels of *SIRT5* (mitochondrial/cytoplasmic) and *SIRT6* (nuclear), both class IV sirtuins, showed inverse associations with brain pH in postmortem control brain samples (r = −0.54, *p* = 0.11 and r = −0.73, *p* = 0.016, respectively), consistent with higher expression at lower pH. While these associations, as well as the corresponding expression changes, were not observed in SCZ and BD cases, in SCZ *SIRT5* expression showed a non-significant trend toward a positive correlation with antipsychotic drug use (r = 0.38, *p* = 0.11), whereas *SIRT6* expression exhibited a significant positive correlation with antipsychotic exposure (r = 0.63, *p* = 0.05). The expression of *SIRT7* (cytoplasmic), another member of the sirtuin family, was significantly decreased in psychotic BD (13%, *p* = 0.005) and showed a non-significant reduction in SCZ (6%, *p* = 0.12), with no detectable effect of antipsychotic drug use on its expression levels.

Several HDAC genes, including *HDAC2*, *HDAC3*, *HDAC4*, *HDAC5*, and *HDAC7*, showed a trend toward inverse correlations with brain pH only in control subjects (r = −0.31 to −0.56). In contrast, *HDAC9* exhibited a direct correlation with pH levels overall (r = 0.42, *p* = 0.02), which was more pronounced in control subjects (r = 0.71, *p* = 0.025). Notably, HDAC9 belongs to the class IIa histone deacetylases, which shuttle between the nucleus and cytoplasm and are implicated in neuronal regulation. It also exhibits relatively weak intrinsic deacetylase activity, but recruits active HDACs like HDAC3 [38]. None of the HDAC genes showed significant expression changes in SCZ or BD, with the exception of *HDAC2* (class I), which trended toward decreased expression in SCZ (7.5%, *p* = 0.06). This effect may be related to antipsychotic drug exposure, as *HDAC2* expression was significantly decreased by antipsychotic use (r = −0.58, *p* = 0.007).

Lysine (K)-specific demethylase 3A (*KDM3A*), which mediates transcriptional activation of target genes, was significantly overexpressed in SCZ (34%, *p* = 0.001), but not in BD (17%, *p* = 0.12). *KDM3A* expression exhibited a strong positive correlation with pH in controls (r = 0.85, *p* = 0.002), but an inverse correlation in BD (r = −0.50). In BD, *KDM3A* expression also showed a trend toward a positive association with antipsychotic use (r = 0.55, *p* = 0.10). Additionally, *KDM3A* expression was positively correlated with *TGFB2* in the combined SCZ and BD cohort (r = 0.53, *p* = 0.01). Lysine (K)-specific demethylase 5B (*KDM5B*) was overexpressed in both SCZ and BD (22% in each, *p* = 0.0008 and *p* = 0.004, respectively). Its expression showed a positive, but non-significant correlation with pH in controls (r = 0.47, *p* = 0.16), whereas, similar to *KDM3A*, it exhibited an inverse correlation with pH in BD (r = −0.66, *p* = 0.036). *KDM5B* expression also showed a positive correlation with *TGFB2* in BD (r = 0.57, *p* = 0.08) and SCZ (r = 0.68, *p* = 0.03), as well as in the combined SCZ and BD cohort (r = 0.60, *p* = 0.01), while showing no correlation with antipsychotic use (r = 0.19).

Methyltransferase-like 7A (*METTL7A*) was also overexpressed in both SCZ and BD (both >60%, *p* = 0.008 and *p* = 0.017, respectively). It exhibited an inverse correlation with pH overall (r = −0.37, *p* = 0.044) as well as across the combined SCZ and BD cohort (r = −0.42, *p* = 0.065). *METTL7A* expression showed no correlation with antipsychotic use, but demonstrated a strong positive correlation with *TGFB2* expression overall (r = 0.78, *p* = 0.000001). Functionally, METTL7A is involved in alkyl thiol methylation and lipid metabolism rather than DNA methylation [39].

*EP300*, which encodes a histone acetyltransferase that functions as writer associated with lactylation-mediated transcriptional activation, showed no significant expression changes in SCZ or BD. In control subjects, its expression trended toward a positive correlation with postmortem brain pH (r = 0.46, *p* = 0.18), whereas this relationship was not observed in SCZ or BD. In BD, *EP300* expression showed a significant positive correlation with antipsychotic drug use (r = 0.76, *p* = 0.01). Similarly, *CREBBP* (CBP), a functional coactivator and paralog of EP300, showed no significant expression changes in SCZ or BD either. In controls, it exhibited a trend toward an inverse correlation with pH (r = −0.33, *p* = 0.35), which was absent in SCZ and BD. Notably, *CREBBP* expression was significantly inversely correlated with antipsychotic drug use (r = −0.49, *p* = 0.028), suggesting a potential medication-associated modulation of its expression–pH relationship in disease states.

*DNMT1* showed no significant expression changes in SCZ or BD, but trended toward a positive correlation with pH in control subjects (r = 0.61, *p* = 0.06). It showed an inverse correlation with antipsychotic use overall (r = −0.32, *p* = 0.15), which was more pronounced in SCZ (r = −0.77, *p* = 0.01). However, *DNMT3A* showed a trend toward an inverse correlation with pH in controls (r = −0.40, *p* = 0.25) and a positive correlation with antipsychotic use across SCZ and BD (r = 0.53, *p* = 0.11, two-tailed), suggesting a potential medication-associated modulation of its relationship with pH across disease states. *DNMT3B* showed no significant associations with pH, diagnosis, or antipsychotic use.

*DNMT3L* showed a modest, but significant decrease in expression in SCZ (10%, *p* = 0.02) and trended toward an inverse correlation with pH in controls (r = −0.34, *p* = 0.25). In addition, *DNMT3L* showed a trend toward a positive correlation with *TGFB2* expression in controls (r = 0.47, *p* = 0.08, one-tailed), but an inverse correlation in the combined SCZ and BD cohort (r = −0.50, *p* = 0.03). It also showed a significant inverse correlation with antipsychotic use, particularly in BD (r = −0.69, *p* = 0.027). Notably, DNMT3L is not an active DNMT, but acts as a cofactor/activator and activates DNMT3A and DNMT3B.

None of the DNA demethylation genes (e.g., *TET1–3*) exhibited significant expression changes in SCZ or BD, despite increases in *TET1* expression of 26% in SCZ and 66% in BD. Notably, in control subjects, *TET2* showed a trend toward a positive correlation with pH (r = 0.45, *p* = 0.18), while *TET3* exhibited a significant positive correlation (r = 0.70, *p* = 0.025). Correlation analyses revealed no significant associations between *TET* gene expression and antipsychotic drug use.

### 2.5. Effects of Lactic Acid on TGFB2 Expression in iPSC-Derived Astrocytes and Neurons

**Figure 5:**
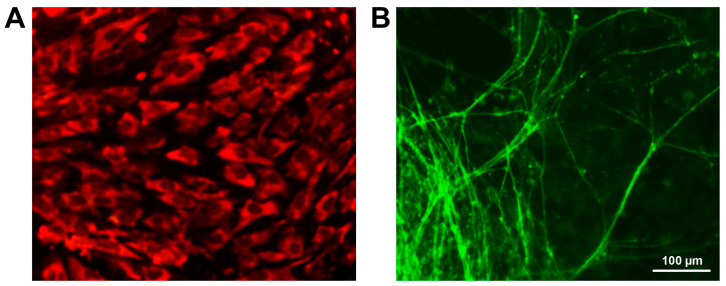
Differentiation of iPSC-derived NSCs into astrocytes and neurons. (**A**) Differentiation of NSCs into astrocytes after 2 weeks, as described in the Materials and Methods section. (**B**) Differentiation of NSCs into neurons. GFAP and MAP2 primary antibodies, together with red and green Alexa Fluor secondary antibodies, were used for immunohistochemical staining of astrocytes and neurons, respectively.

**Figure 6:**
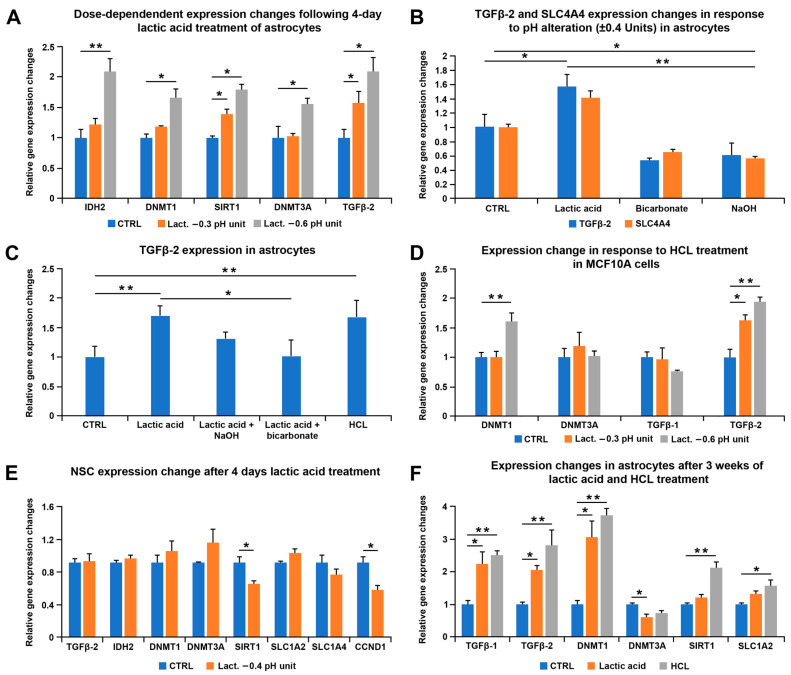
Influence of pH alteration on TGFB2 expression in iPSC-derived astrocytes, MCF10A cells, and NSCs. (**A**). Appropriate doses of lactic acid were used to decrease the pH of the culture medium by 0.3 (−0.3) and 0.6 (−0.6) units (from pH 6.8 to 6.5 and 6.2, respectively). A dose-dependent increase in gene expression was observed in iPSC-derived astrocytes, particularly at the higher dose. (**B**). Next, we selected an intermediate effective dose of lactic acid, as well as bicarbonate or NaOH, to modify the pH of the culture medium by approximately ±0.4 pH units. Lactic acid treatment increased the expression of *TGFB2* and *SLC4A4*, whereas bicarbonate or NaOH treatment decreased the expression of these genes. (**C**). Correction of pH to control levels using bicarbonate or NaOH in combination with lactic acid treatment significantly reduced *TGFB2* expression. (**D**). Similar to lactic acid treatment, HCl treatment also increased *DNMT1* expression at higher doses, whereas the expression levels of *DNMT3A* and *TGFB1* remained unchanged. In contrast, *TGFB2* exhibited a dose-dependent increase in expression. (**E**). NSCs did not exhibit similar changes to those observed in astrocytes following a four-day lactic acid treatment. (**F**). The increased expression pattern was sustained following three weeks of HCl and lactic acid treatment, which induced a reduction of approximately 0.4 (−0.6) pH units relative to control astrocytes. One and two asterisks (*) denote *p* < 0.05, *p* < 0.01, respectively.

Several studies, including meta-analytic evidence, encompassing more than 10 independent datasets have reported that decreased brain pH in SCZ and BD is associated with elevated brain lactate level [23,24,25]. Interestingly, a mechanistic study in metabolic disease further demonstrated that lactate can induce *TGFB2* expression, an effect that is attenuated by anti-lactate interventions [30]. Therefore, we sought to examine whether similar phenomena to the metabolic disease would exist in the psychiatric diseases using renewable cellular systems as a potential avenue for drug discovery. We hypothesized that lactic acid or HCL (i.e., lower pH) could also induce the expression of *TGFB2* and *TGFB2*-correlated genes in iPSC-derived NSCs, neurons, astrocytes and brainoids. To test this, we differentiated iPSCs to NSCs and then to astrocytes and neurons (Figure 5) as described in Materials and Methods. Next, iPSC-derived NSCs, neurons, and astrocytes along with multiple cell lines were treated with two incremental doses of HCl or lactic acid for four days. These experiments revealed a dose-dependent increase in *TGFB2* expression, along with several genes involved in epigenetic regulation, in iPSC-derived astrocytes (Figure 6A). In contrast, no similar changes were observed in neurons, with the exception of *IDH2*, which showed a 30% increase in expression (*p* = 0.03, SD = 0.04), and *DNMT1*, which exhibited a 40% decrease relative to controls (*p* = 0.02, SD = 0.16) in neurons. *TGFB2* expression also showed a trend toward reduction (20%, *p* = 0.06, SD = 0.18), but *SIRT1* and *DNMT3A* exhibited no significant change, with a nearly 20% decrease in expression (*p* = 0.1, SD = 0.1 and *p* = 0.4, SD, 0.16, respectively) under the same conditions in neurons.

After establishing that lactic acid induces dose-dependent increases in *TGFB2* and related genes, we decided to select the middle effective dose of lactic acid (corresponding to a 0.4-unit reduction in culture medium pH) for subsequent experiments. Under this condition, and consistent with observations in postmortem brain samples, several genes exhibited altered expression in astrocytes. In addition to significantly increased expression of *IDH2*, *DNMT1*, *SIRT1* and *SLC4A4* (Figure 6A,B), *MAOA* exhibited a 250% increase (*p* = 0.035, SD = 0.40), and *IDH1* a 90% increase (*p* = 0.05, SD = 0.29) following lactic acid treatment. Furthermore, *TGFB1* and *SLC1A2* expression showed a significant increase of 28% (*p* = 0.004) and 32% (*p* = 0.04), respectively, following lactic acid treatment.

We discontinued lactic acid treatment after four days and observed that *TGFB2* and *DNMT1* expression returned to baseline levels in astrocytes. To examine whether increased pH has an opposite effect, astrocytes were treated with bicarbonate or NaOH to raise the culture medium pH by 0.4 units. As shown in Figure 6B, both bicarbonate and NaOH treatment decreased the expression of *TGFB2* and *SLC4A4*. Additionally, correction of pH to control levels using bicarbonate or NaOH significantly reduced *TGFB2* expression, bringing it close to that observed in control astrocytes, while HCL also increased *TGFB2* expression (Figure 6C). We observed comparable effects in other independent cellular systems such as MCF10A, an immortalized mammary epithelial cell line (Figure 6D), as well as HEK293 cells and fibroblasts, but not in cancer cell lines (Supplementary Figure S1). In NSCs, while no changes similar to those observed in astrocytes were detected following a four-day lactic acid treatment, we observed a significant reduction in the expression of *SIRT1* and *CCND1* of approximately 30% and 40%, respectively (Figure 6E). Therefore, the effects of pH and acidification on *TGFB2* expression may be cell-context dependent rather than universally conserved across all cell types. To determine whether the lactic acid–induced increase in *TGFB2* expression is sustained under prolonged exposure, astrocytes were treated for three weeks. Under these conditions, the expression levels of *TGFB1*, *TGFB2*, *DNMT1*, *SIRT1* and *SLC1A2* remained significantly elevated, indicating a sustained effect of long-term lactic acid treatment (Figure 6F).

In gene expression analyses of iPSC-derived astrocytes, *CA1* showed no detectable expression, consistent with its expected minimal or absent expression in brain tissue; however, low-level expression was detected in neurons. *CA2*, a cytosolic carbonic anhydrase predominantly expressed in oligodendrocytes, unexpectedly showed significantly decreased expression under acidic conditions (*p* = 0.02, SD = 0.07), similar to observations in postmortem brains of control subjects (r = 0.56, *p* = 0.09), although this relationship was not significant in SCZ (r = 0.35) or BD (r = −0.14). Notably, *CA2* expression was not significantly altered in postmortem brain samples from SCZ or BD patients, and antipsychotic drug use showed an inverse trend with *CA2* expression in SCZ (r = −0.59, *p* = 0.07) and a positive trend in BD (r = 0.60, *p* = 0.07). *CA5A* showed limited expression in astrocytes, consistent with its pattern in postmortem brain tissue, where it also exhibited a trend toward an inverse correlation with pH in controls only (r = −0.55, *p* = 0.09).

### 2.6. Effects of Gene Expression Alterations in Response to Lactic Acid Treatment of Brainoids

**Figure 7:**
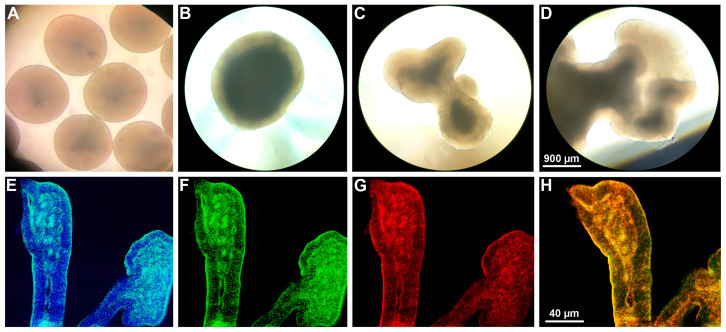
iPSC-derived brainoids as described in the Materials and Methods section. (**A**) Spheroids after 2 days in culture; (**B**) one spheroid after 1 week. (**C**) Cortical formation after 2 weeks; (**D**) portion of a brain organoid measuring 0.7 mm in size. Scale bars represent 900 µm. (**E**) Region of an organoid with cortical formation stained with a MAP2 primary antibody and an Alexa Fluor green secondary antibody, indicating neurons, while DAPI staining marks cell nuclei. (**F**) MAP2 staining alone. (**G**) Staining with a GFAP primary antibody and a red Alexa Fluor secondary antibody labeling astrocytes. (**H**) Overlay of MAP2 and GFAP staining.

The ability to efficiently produce disease-specific human cell models consistently at scale opens greater opportunities to study neurological disorders in vitro. Thus, the multicellular 3D brainoids recreating complex neural interactions are likely a better platform compared to 2D culture systems. Figure 7 illustrates the step-by-step generation of 3D brainoids and the validation of major brain cell identities, as described in the Materials and Methods section. We performed gene expression analysis following 5 days of lactic acid exposure, resulting in an approximately 0.4-unit decrease in medium pH, as described above. Under these conditions, *TGFB2* expression was significantly increased by more than twofold (fold change = 2.15, *p* = 0.012, SD = 0.18). The direction of change for most of the other candidate genes was consistent with that observed in astrocytes and/or postmortem brain samples, as shown in Table 1. However, in contrast to postmortem brains, which showed no expression change in response to pH, the expression of *SLC16A7* (primarily an inward lactate transporter) and SLC16A1 (also an inward lactate transporter in addition to being a short-chain fatty acid transporter) exhibited significant increases following lactic acid treatment, by 75% (*p* = 0.03) and 360% (*p* = 0.0004), respectively. Among carbonic anhydrase genes, the expression of *CA1* and *CA5A*, which showed a trend toward inverse correlation with pH in postmortem control brains (r = −0.49 and r = −0.55, respectively) and a trend toward correlation with *TGFB2* expression in SCZ and BD (r = 0.39, *p* = 0.09), were also unexpectedly reduced following lactic acid treatment in brainoids, by 81% (*p* = 0.08) and 60% (*p* = 0.027). Similarly, the expression of CXCR4, which is involved in neuroinflammation and astrocyte activation [40], was increased following lactic acid treatment in brainoids (2.3-fold, *p* = 0.04), whereas in control postmortem brains it showed a positive correlation with pH (r = 0.61, *p* = 0.06). In contrast, we observed decreased expression of *DTNBP1* (55%, *p* = 0.04) following lactic acid treatment but in postmortem brain of controls, *DTNBP1* exhibited an inverse correlation with pH (r = −0.61, *p* = 0.06). Although this was not observed in SCZ and BD, there was a positive correlation with antipsychotic drug use (r = 0.34, *p* = 0.07), particularly in SCZ (r = 0.71, *p* = 0.02), which may attenuate or obscure its potential inverse correlation with pH in SCZ and BD. *OCT4* was another gene showing decreased expression in brainoids following lactic acid treatment (55%, *p* = 0.025). However, in postmortem brains (albeit at very low expression levels), *OCT4* also showed a trend toward a direct correlation with pH overall (r = 0.31, *p* = 0.09) and a significant direct correlation in control subjects (r = 0.69, *p* = 0.03). Additionally, *EN2*, which trended toward an inverse correlation with pH in controls (r = −0.55, *p* = 0.09), showed the same trend in brainoids (increased 35% increase, *p* = 0.11). A similar directionality of expression changes consistent with that observed in postmortem brain samples from controls or the overall cohort was also identified for *NURR1*, *CCND1*, *MAOA*, *TH*, *IDH2*, *TNFA*, *IL13RA2*, *SLC1A2*, and *DNMT3A*. Table 1 summarizes the expression profiles of the investigated genes in brainoids.

In respect of genes important in neurotransmitter synthesis or neurotransmission, *TPH2* (a serotonin synthase) exhibited a 250% increase (*p* = 0.0005), *TH* (tyrosine hydroxylase, involved in dopamine synthesis) a 50% increase (*p* = 0.08) and *HTR2A* a 290% increase (*p* = 0.01) in expression in brainoids following lactic acid treatment. In contrast, we observed decreased expression of *NURR1* (80%, *p* = 0.007) following lactic acid treatment.

### 2.7. DNA Methylation Alterations in Response to Lactic Acid Treatment in iPSC-Derived Astrocytes, Neurons and Brainoids

**Figure 8:**
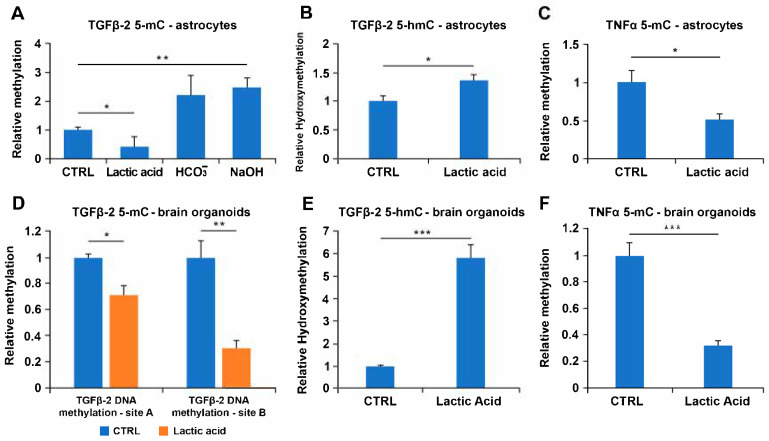
Influence of pH alteration and lactic acid treatment on promoter DNA methylation in iPSC-derived astrocytes, neurons, and brainoids. (**A**). Astrocytes were treated for four days with lactic acid, bicarbonate (HCO_3_^−^), or NaOH to modify the pH of the culture medium by approximately ±0.4 pH units. Lactic acid treatment decreased *TGFB2* promoter DNA methylation, whereas bicarbonate and NaOH treatment (increasing pH by 0.4 units) increased DNA methylation of the *TGFB2* promoter region. (**B**). Lactic acid treatment was associated with increased *TGFB2* promoter hydroxymethylation (5-hmC) at site B in astrocytes. (**C**). Promoter DNA methylation of *TNFA* was also reduced following lactic acid treatment in astrocyte. (**D**). In brainoids, lactic acid treatment decreased *TGFB2* promoter DNA methylation at both site A and site B; however, the reduction at site B, which is located closer to the coding region, was more prominent. (**E**). In brainoids, lactic acid treatment increased *TGFB2* promoter 5-hmC also level. (**F**). Similar to astrocytes, *TNFA* promoter DNA methylation was also decreased following lactic acid treatment in brainoids. One, two, and three asterisks (*) denote *p* < 0.05, *p* < 0.01, and *p* < 0.001, respectively.

As shown in Figure 8, lactic acid treatment decreased *TGFB2* promoter DNA methylation in astrocytes, whereas bicarbonate and NaOH treatments had opposite effects (Figure 8A). At the same time, *TGFB2* promoter hydroxymethylation (5-hmC) was increased following lactic acid treatment in astrocytes (Figure 8B). Similarly, DNA methylation of *TNFA* promoter in astrocytes decreased to half following lactic acid treatment in astrocytes (Figure 8C). In neurons, there was a trend toward a mild increase in *TNFA* promoter methylation (33%, *p* = 0.09, SD = 0.1) following lactic acid treatment, while no change in *TGFB2* promoter DNA methylation was observed (1, vs. 1.08, *p* = 0.57, SD = 0.13). The promoter region of *SLC1A2* (an astrocytic gene with around sevenfold higher DNA methylation in neurons compared to astrocytes) exhibited a nearly 25% increase in DNA methylation following lactic acid treatment in neurons (*p* = 0.02, SD= 0.05). In contrast, *MECP2* (highly expressed in neurons and with baseline promoter methylation of ~20% vs. 60% in astrocytes) showed >20% decrease in DNA methylation (*p* = 0.02, SD = 0.04) following lactic acid treatment. We found no significant changes in *IFI16*, *GRID2*, *SLC16A1*, or *SLC16A7* methylation levels in astrocytes or neurons.

In respect of brainoids, Table 2 summarizes DNA methylation status and expression changes of more than 20 brain functional genes, of which approximately half exhibited altered expression in postmortem brain samples of patients with SCZ and/or BD. We also included twelve genes that showed no expression changes in postmortem brain tissue to evaluate whether DNA methylation patterns in brainoids recapitulate pH-associated gene expression profiles observed in postmortem samples (i.e., serving as negative controls). The results indicate that the absence of expression changes is generally associated with a lack of detectable DNA methylation alterations in brainoids (Table 2).

Similar to postmortem brain samples and astrocytes, *TGFB2* DNA methylation was reduced by lactic acid (Figure 8D), while its 5-hmC level was increased (Figure 8E). Likewise, *TNFA* promoter DNA methylation was also decreased in brainoids (Figure 8F). As shown in Table 2, promoter DNA methylation of *MAOA* (which shows increased expression in postmortem brain samples) was decreased (42%, *p* = 0.012), while its promoter 5-hmC was increased (85%, *p* = 0.025) in brainoids following lactic acid treatment. However, *MECP2*, which showed no expression change in SCZ and BD postmortem brain samples but exhibited direct correlation with pH (r = 0.65, *p* = 0.04) and inverse correlation with *TGFB2* expression (r = 0.63, *p* = 0.05) in control subjects, exhibited increased promoter DNA methylation following lactic acid treatment, which is in line with its direct expression correlation with pH in postmortem brain samples.

Remarkably, as shown in Table 2, in addition to *TGFB2*, *TNFA*, and *MAOA*, most of the other genes, including *SLC1A2*, *SLC1A3*, *SLC16A1*, *RELN*, *IFI16*, *EN2*, *AUTS2*, *TINF2*, *MBD4*, and *CXCR4*, exhibited increased DNA hydroxymethylation in brainoids, suggesting that lactic acid tends to promote DNA hydroxymethylation in brain cells.

## 3. Discussion

Epidemiologic and genetic studies indicate that SCZ and BD are mental health diseases with complex and multifactorial origin influenced by both genetic and environmental factors, which contribute to dysregulation of hundreds of genes in the brain and other tissues. Our previous studies provided evidence that abnormal DNA methylation of the regulatory regions of several genes, including *TGFB2*, plays a significant role in defining the gene expression patterns involved in the pathogenesis of SCZ and BD. Other studies, including a meta-analysis, also concluded that TGFβ is a disease state marker for SCZ, as its expression increased in first-episode psychosis and acutely relapsed patients, but normalized by antipsychotic treatment. As a recent mechanistic study reported that lactic acid increases *TGFB2* expression and anti-lactate drugs inhibit its effect, other studies have shown that an increase in brain lactate level is the leading cause of decreased brain pH reported in SCZ and BD. While this was supported by a meta-analysis of a dozen human studies, as well as in drug-naïve animal models of psychiatric diseases, we found that reduced brain pH in SCZ and BD is associated with increased expression of *TGFB2* and many other genes exhibiting direct correlation with *TGFB2* expression.

Ingenuity pathway analysis (IPA) and Spearman correlation analysis of our expression microarray data of postmortem brain samples from SCZ and BD patients versus controls revealed that *TGFB2* is likely a key driver of dysregulation of >75% genes (in particular astrocytic genes) in SCZ and BD. Furthermore, as there was an inverse correlation between *TGFB2* expression and brain pH (r = −0.54, *p* = 0.002), a large fraction of TGFB-related genes (e.g., *TGFBR3*, *SMAD1*, *BMP7* and *BMPR1B*) were upregulated and inversely correlated with brain pH. Our complementary gene expression and DNA methylation analysis of larger sets of postmortem brain samples (35/group) revealed that increased *TGFB2* expression in SCZ and BD is associated with its promoter DNA hypomethylation [31]. Furthermore, our analysis of postmortem brain samples along with other studies suggest that a decrease in brain pH connected to an increase in brain lactate level and increase *TGFB2* expression is likely a common feature in major mental disorders [24,25,31]. Therefore, we decided to test the hypothesis that lactate-mediated brain gene dysregulation associated with reduced pH level may be involved in SCZ and BD pathophysiology. Considering ethical and practical obstacles, which prevent molecular research on human brains, we sought to develop a model system that recapitulated the features of SCZ and BD, which may assist in finding new preventive or therapeutics for the disease state. Here, we studied the impact of low pH (using lactic acid or HCL) and potential normalizing effects by higher pH (using NaOH or bicarbonate) in iPSC-derived neurons, astrocytes, and brainoids on the expression and promoter DNA methylation of *TGFB2* and some key correlated dysregulated genes.

We found strong evidence that lactic acid increases *TGFB2* expression by inducing its promoter DNA hypomethylation in iPSC-derived astrocytes and brainoids. While a landmark metabolic study in mice revealed that anti-lactate drugs neutralize lactate effects of *TGFB2* expression, we found that bicarbonate and NaOH could decrease *TGFB2* expression and increase its promoter DNA methylation. Furthermore, while our analysis of postmortem brain samples revealed that many genes along with *TGFB2* exhibit increased gene expression as brain pH is decreased, we showed that the expression of those correlated genes is also increased by lactic acid treatment both in iPSC-derived astrocytes and brainoids, but not markedly in neurons. Other studies also uncovered that pH alteration affects mostly astrocytic genes, as astrocytes do not merely provide structural support, but actively manage the chemical environment of the brain to prevent harmful acidification during intense neuronal activity [29].

Our correlation analysis, together with in vitro studies on astrocytes and brainoids indicates that although *TGFB2* may orchestrate the expression of other dysregulated genes, it might be lactate/pH that affects the expression of many genes along with *TGFB2*. However, further experiments involving *TGFB2* overexpression or suppression may provide additional evidence to clarify whether *TGFB2* plays a primary regulatory role or whether pH-related effects are the predominant driver of the observed gene expression changes. Notably, the use of antipsychotic drugs was associated with a negligible trend for inverse correlation with pH (r = −0.22, *p* = 0.35), suggesting that a small portion of the reduced brain pH might be due to antipsychotic drugs. Among the first 300 genes that exhibited inverse correlation with pH, only 23% trended or exhibited direct correlation with antipsychotic drug use (r > 0.4). However, almost all of the affected genes with an increased expression connected to lower brain pH in SCZ and BD exhibited inverse correlation with pH in controls as well. For example, in the overall dataset (30 postmortem brain samples), among the top 100 genes exhibiting an inverse correlation with pH, 56% of them in controls also showed a similar trend (r < −0.4) or a significant inverse correlation (r < −0.61; 17%) with pH, while none of the remaining genes displayed a positive correlation with brain pH. This supports that acidic brain pH is a driver of the increased gene expression, not only in the disease state but also in the brain of control subjects. Among these 100 genes, 10% showed a trend (r > 0.4) and 23% showed a significant positive correlation (r > 0.44) with antipsychotic drug use. These findings suggest that the use of antipsychotic drugs (by increasing brain pH) may abolish the actual inverse correlation of a significant number of genes in SCZ and BD patients, which may be present in drug-naïve patients.

Our findings further indicate that in addition to *TGFB2*, lactic acid treatment of brainoids and astrocytes (but not neurons) induces DNA hypomethylation of some key genes exhibiting increased expression in postmortem brain samples (e.g., *TNFA* and *MAOA*). Notably, a subset of these genes also exhibits increased promoter hydroxymethylation, a modification generally linked to elevated gene expression. From a mechanistic point of view, it is likely that the reduced brain pH may lead to DNA hydroxymethylation and demethylation (mediated in part by *IDH1* and *IDH2* increased expression) and upregulation of many genes in SCZ and BD, which predominantly exhibit increased expression in the disease states. While IDH1 and IDH2 enzymes increase a-ketoglutarate, which increase TET enzyme activity, leading to hydroxymethylation [37], we observed a significant increase in the expression of *IDH1* (*p* = 0.045) and *IDH2* (*p* = 0.04) genes following lactic acid treatment. DNA hydroxymethylation of several genes was also increased in brainoids, which mostly exhibited increased expression (e.g., *TGFB2*, *SLC16A1*, *SLC1A2*, *SLC1A3*, *EN2* and *CXCR4*). On the other hand, we found increased expression of *DNMT1* only in astrocytes (which could be reactive) and DNA hypermethylation of a small number of genes linked to brain function (e.g., *NURR1*, *TINF2*, *AUTS2* and *MBD4*) in brainoids. This could be due to the lack of increased expression of DNMT1 in brainoids, consisting of both neurons and astrocytes. As mentioned in the Results section, in contrast to astrocytes, iPSC-derived neurons exhibited reduced expression of *DNMT1* following lactic acid treatment, and hence the neuronal contribution likely dampens the astrocyte-driven increase in the organoid setting.

Among other interesting genes, *CCND1* was the gene with the most significantly reduced expression in SCZ (44%, *p* = 0.0004) and to a lesser extent in BD (21%, *p* = 0.04) in postmortem brain samples. *CCND1* exhibited a trend for inverse correlation with pH in SCZ and control subjects (r = −0.41 and −0.46, respectively), but not in BD (r = 0.27). Therefore, the reduced brain pH in SCZ could likely induce its expression. This was observed in brainoids (30% increased by lactic acid, *p* = 0.01), suggesting that its original expression in postmortem brains of patients with SCZ might be even lower. However, a longer period of antipsychotic drug treatment was associated with its decreased expression in BD (r = −0.53, *p* = 0.11), but not in SCZ (r = −0.27). The disease-related reduced *CCND1* level may therefore reflect the net of opposing influences (pH-driven upregulation vs. antipsychotic-driven suppression). Interestingly, in NSCs, acidic pH decreased *CCND1* expression, suggesting that lower brain pH during early developmental periods may contribute to its inherent downregulation in SCZ and BD.

*DTNBP1* is another interesting SCZ-related gene that exhibited an inverse correlation with pH in control subjects (r = −0.61, *p* = 0.05), but not significantly in SCZ or BD. Since there was a direct correlation with the use of antipsychotic drugs (r = 0.34), particularly in SCZ (r = 0.71, *p* = 0.02), the disease-state pH–*DTNBP1* relationship is likely attenuated by antipsychotic treatment. Consistent with this, another study reported that antipsychotic drugs attenuate downregulation of *DTNBP1* expression associated with its promoter DNA hypermethylation in SCZ and BD [41].

Regarding the innate mechanisms that regulate pH, we also observed disparity between controls and patients. For example, in control subjects we found several *CA* genes (e.g., *CA1*, *CA5A*, *CA3*, *CA9*, *CA12*, and *CA13*) trended toward or exhibited significant upregulation in response to decreased brain pH to produce more bicarbonate ions (HCO_3_^−^) to stabilize brain pH. This compensatory pattern, however, was not observed in SCZ or BD despite their lower brain pH. Ironically, *CA2* expression trended toward direct correlation with pH in controls (*p* = 0.56, r = 0.09) and less prominently in SCZ (r = 0.36), where antipsychotic drugs reduced its expression (r = −0.59, *p* = 0.07), likely attenuating any underlying *CA2*–pH relationship, whereas in BD, antipsychotics increased its expression (r = 0.60, *p* = 0.07). This discordance, together with the relatively modest *CA2* signal in controls, may reflect limited sample size and requires confirmation in larger cohorts. Based on our analysis, antipsychotic drugs could also increase the expression of *CA1* and *CA9* in SCZ (r = 0.67, *p* = 0.034 and 0.75, *p* = 0.012, respectively), but not in BD (r = ~0). These preliminary data indicate that as several key genes involved in pH regulation remained dysregulated in SCZ and BD, antipsychotic drugs may provide limited benefits. Nevertheless, the level of expression of these genes was 16 times less than *CA2* (log2 of 5 vs. 9.5), presenting much limited beneficial effects. Notably, the expression of *CA14* with fivefold less expression compared to *CA2* also trended toward increased expression by antipsychotic drugs in BD (r = 0.52, *p* = 0.12), but reduced expression in SCZ (r = −0.89, *p* = 0.0005), indicating no beneficial effects in pH regulation, at least in SCZ.

## 4. Materials and Methods

### 4.1. Samples and Cell Lines

In this study, we analyzed a total of 105 DNA and RNA samples from the frontal cortex of postmortem brains of patients with SCZ and BD, as well as matched controls (*N* = 35 per group). Samples were obtained from the Stanley Medical Research Foundation (SMRF) in coded form, and the investigators did not have access to subject identifiers. The study was determined to qualify for Exempt Category 4 by the Institutional Review Board (IRB) at the Boston Medical Center (Protocol H-30274; approved 23 April 2011). Brain pH was measured during tissue processing, with an overall range of 5.76–7.03, including 6.00–7.03 in controls, 5.90–6.93 in SCZ, and 5.76–6.97 in BD. The SMRF dataset also provided information on lifetime antipsychotic exposure for SCZ and BD subjects, as well as postmortem interval, refrigeration interval, and additional demographic and clinical covariates for both patients and control subjects. Lifetime antipsychotic exposure was quantified as cumulative antipsychotic intake expressed in fluphenazine-equivalent milligrams up to the time of death, ranging from 0 to 400,000 mg, and is hereafter referred to as antipsychotic drug use/exposure or extent of antipsychotic drug use.

Two human control iPSC lines purchased from the Simons Foundation (SV0001455 and SS0013111), as well as one additional iPSC line from foreskin fibroblast-derived cell line (iPS(foreskin)-3; WiCell Research Institute, Madison, WI, USA; cat. #WB0002) for which no IRB or ESCRO review was required, were used in this study. Primarily hiPSCs from the Simons Foundation were differentiated into neural stem cells (NSCs), which were subsequently differentiated into neurons and astrocytes as previously described [42]. Briefly, hiPSCs were cultured in StemFlex medium (Thermo Fisher, Waltham, MA, USA, Cat# A3349401) supplemented with 5 µM ROCK inhibitor (Thermo Fisher, Cat# A26445-01) in 12-well plates coated with Geltrex (Thermo Fisher, Cat# A1569601) and maintained at 37 °C in a humidified incubator with 5% CO_2_. The medium was replaced after 24 h and subsequently changed every other day. After three passages, hiPSCs were differentiated into NSCs using a neural induction medium consisting of KnockOut DMEM/F-12 supplemented with Gibco StemPro Neural Supplement, bFGF, EGF, and GlutaMAX-I (Thermo Fisher, Cat# A1647801), along with 1% antibiotic–antimycotic solution (Thermo Fisher, Cat# 15240062), on Geltrex (Thermo Fisher Cat# 12760)-coated dishes.

Following three passages, NSCs were differentiated into neurons using neurobasal medium supplemented with B-27 (Thermo Fisher, Cat# 17504044) on dishes coated with laminin (Thermo Fisher Cat# 23017) and poly-L-ornithine (MilliporeSigma, Burlington, MA, USA, Cat# P3655) or into astrocytes using DMEM supplemented with N2 (Thermo Fisher, Cat# A1370701) and 0.5% FBS on Geltrex-coated dishes. After two weeks of differentiation, acquisition of neuronal and astrocytic identity was confirmed by immunocytochemistry. Cells were fixed, permeabilized, and incubated with blocking buffer, followed by overnight incubation with primary antibodies against MAP2 (Invitrogen, Waltham, MA, USA, Cat# MA1-25043) for neurons and glial fibrillary acidic protein (GFAP; Abcam, AB68428) for astrocytes. This was followed by incubation with appropriate Alexa Fluor-conjugated secondary antibodies (e.g., Alexa Fluor 488 for neurons and Alexa Fluor 594 for astrocytes), as previously described [42].

A 4-day treatment with lactic acid or HCl was initiated at week 3 of differentiation, in duplicate, when cultures reached approximately 50% confluency. The treatments were applied to induce controlled pH reductions of approximately 0.3 and 0.6 pH units in a 2D culture system. In addition to hiPSC-derived NSCs, neurons, and astrocytes, several other cell lines, including MCF10A (human mammary epithelial cell line from ATCC, catalog number: CRL-10317), adult human dermal fibroblasts (HDFa; ATCC PCS-201-012, Manassas, VA, USA), human embryonic kidney cells (HEK293; ATCC CRL-1573, Manassas, VA, USA), as well as MCF10CA1h breast cancer (MIII; Cellosaurus CVCL_6683, from Barbara Ann Karmanos Cancer Institute, Detroit, MI, USA), HCT116 *SMAD4+/+*, a human colorectal carcinoma cell line [(a kind gift from Dr. Bert Vogelstein (Johns Hopkins)], and MDA-MB-231 breast cancer (ATCC HTB-26, Manassas, VA, USA) cell lines were treated with lactic acid or HCl to reduce controlled pH by 0.3 and 0.6 pH units. Following these preliminary studies, astrocytes were also treated with bicarbonate or NaOH to induce controlled pH increases of approximately 0.4 pH units over 4 days in duplicate cultures, with daily medium replacement.

In addition to the 2D culture system, brain cortical organoids were generated with minor modifications to the method described by Gabriel et al. [43] to assess the effects of pH alterations in a 3D brain model. Briefly, using two iPSC lines (the human foreskin fibroblast-derived cell line iPS(foreskin)-3 obtained from the WiCell Research Institute and SV0001455 cells obtained from the Simons Foundation) human brainoids were generated through the formation of human embryoid bodies (hEBs) from hiPSCs using an agarose-based microwell aggregation platform previously developed and validated by our research group [44]. Microwell arrays were pre-equilibrated overnight at 37 °C under 5% CO_2_ in EB differentiation medium consisting of a 1:1 mixture of IMDM and Ham’s F-12 nutrient mixture (Invitrogen) supplemented with 5% fetal bovine serum (Invitrogen), 1% (*v*/*v*) insulin–transferrin–selenium A (Invitrogen), 55 μM monothioglycerol (Sigma-Aldrich, Burlington, MA, USA), 100 U mL^−1^ penicillin, and 0.1 mg mL^−1^ streptomycin (Invitrogen). Single-cell-dissociated hiPSCs were subsequently seeded into the microwell arrays at a density of 35,000 cells per microwell, enabling the formation of hEBs within 24 h. Subsequently, hEBs were transferred into a 35 mm petri dish using a cut 200 µL pipette tip and maintained for two days in neurosphere medium composed of DMEM/F12 (48.4% *v*/*v*), neural basal medium (48.4% *v*/*v*), N2 supplement (0.4×), B27 (0.2×), GlutaMAX (1×), MEM (0.5×), insulin (0.2755 µM), SB431542 (2.5 µM), penicillin–streptomycin (100 U/mL), β-mercaptoethanol (5 µM), and 0.1% Matrigel [43], plus 5 µM ROCK inhibitor.

At day 3, 12 embryoid bodies were transferred into a 35 mm petri dish containing 3 mL of neurosphere medium and incubated for an additional 3 days at 37 °C and 5% CO_2_ on an orbital spinner at 120 rpm. On day 4 of spinner culture, the medium was replaced with fresh medium consisting of DMEM/F12 (48.4% *v*/*v*), neural basal medium (48.4% *v*/*v*), N2 supplement (0.4×), B27 (0.2×), GlutaMAX (1×), MEM (0.5×), dorsomorphin (0.5 µM), insulin (0.2755 µM), SB431542 (5 µM), penicillin–streptomycin (100 U/mL), and β-mercaptoethanol (5 µM). The culture medium was partially replaced every 2 days (75% medium exchange) for 8 weeks, during which brainoids developed a cortical-like morphology with diameters of approximately 3–5 mm. Subsequently, eight organoids were placed in each well of ultralow-attachment 12-well plates in duplicate, and experimental pH modulation was performed by the dropwise addition of lactic acid to reduce the culture medium pH by 0.4 units. Although medium changes were performed every 2 days, pH levels were monitored daily and adjusted as needed to maintain the target pH for up to 5 days between adjustments. Five organoids from each well were used for RNA/DNA extraction, and the remaining three were fixed in 4% paraformaldehyde for 1 h, then stored in PBS at 4 °C for subsequent for subsequent immunohistochemistry analysis.

### 4.2. RNA/DNA Extraction, Gene Expression, and DNA Methylation Analysis

Total RNA and DNA were isolated from samples using TRIzol reagent in combination with a Zymo Research RNA/DNA isolation kit (Irvine, CA, USA, Cat# R2080). RNA quantity and quality were assessed using a NanoDrop One spectrophotometer (Thermo Scientific, Waltham, MA, USA). Complementary DNA (cDNA) was synthesized using the Invitrogen cDNA synthesis kit according to the manufacturer’s instructions (Cat#18090050). Relative gene expression levels were then quantified by quantitative real-time PCR (qRT-PCR) using Applied BioSystems Power SYBR Green Master Mix (Carlsbad, CA, USA, Cat# 4367659). Primer sequences are listed in Supplementary File S2 (Tables S1 and S2). Gene expression was calculated using the ΔΔCt method and normalized to β-actin (ACTB) as the internal control.

For DNA methylation analysis, enzymatic digestion of extracted genomic DNA was performed using HpaII and MspI restriction enzymes in combination with quantitative PCR (qPCR) to assess levels of 5-methylcytosine (5-mC) and 5-hydroxymethylcytosine (5-hmC), respectively, at promoter regions of candidate genes, as previously described [42]. Briefly, 1 µg of genomic DNA from each sample was treated with uridine diphosphoglucose (UDP-glucose) and T4 phage β-glucosyltransferase (T4-BGT) in a 150 µL reaction mixture and incubated overnight. Following glucosylation, the reaction mixture was aliquoted into three separate PCR tubes for downstream restriction enzyme digestion and qPCR analysis. One aliquot was digested with HpaII, another with MspI, and a third aliquot was left undigested and incubated at 37 °C overnight. Following restriction digestion, samples were treated with proteinase K for 30 min at 50 °C, followed by enzyme inactivation at 80 °C for 20 min.

Notably, HpaII does not cleave CCGG sites containing 5-methylcytosine (5-mC). MspI, in contrast, cleaves CCGG sites regardless of 5-mC status, but is sensitive to 5-hydroxymethylcytosine (5-hmC) following T4 phage β-glucosyltransferase (T4-BGT) and UDP-glucose treatment, which converts 5-hmC to glucosyl-5-hmC and thereby protects these sites from MspI-mediated digestion. Following qPCR analysis using gene-specific primers, undigested DNA was used as an internal reference for normalization of both HpaII- and MspI-digested samples. Relative differences in amplification were used to infer levels of 5- 5-mC and 5-hmC, respectively, according to the EpiMark 5-hmC and 5-mC analysis kit protocol (New England BioLabs, Ipswich, MA, USA, Cat# E3317S).

It is important to note that since this method only detects methylation at CCGG sites, we designed primer pairs to target genes promoter regions encompassing multiple CCGG sites; however, a few primer pairs encompassed only one or two CCGG sites (Supplementary Table S2). From the functional point of view, 5-mC is generally associated with transcriptional repression, but 5-hmC is linked to transcriptional activation [45,46]. Since most of other methods for gene-specific DNA methylation analyses, such as pyrosequencing, bisulfite sequencing, and qMSP (quantitative methylation-specific PCR), and Illumina whole-genome DNA methylation profiling cannot discriminate 5-hmC from total DNA methylation, we used this method for quantitative analysis of both 5-mC and 5-hmC with different functional features. The ΔΔC_T_ method was used to quantify 5-hmC levels normalized with the C_T_ of uncut DNA [31]. For quality control, PCR products were further analyzed by acrylamide gel electrophoresis, in addition to melt curve analysis, to verify product specificity and confirm the expected amplicon size.

### 4.3. Statistical Analysis

A detailed statistical analysis of the microarray data has been reported previously [31]. In the present study, in addition to two-tailed Student *t*-tests, statistical analyses included Pearson’s correlation, as well as univariate, bivariate, and multivariate approaches, selected according to the structure and distributional characteristics of the data. Parametric tests and large-sample approximations were applied where assumptions of normality and variance homogeneity were satisfied. For datasets with small samples, exact tests, including Fisher’s exact test, were used. All statistical tests were two-sided unless otherwise specified, and α = 0.05 was used as the nominal significance threshold. Where applicable, results are reported with corresponding test statistics and *p*-values. Because gene-level correlations were performed across large gene sets, these analyses should be interpreted as exploratory. For transparency, full nominal *p*-values and FDR-adjusted values are provided in Supplementary File S1 (Sheet S3). Quantitative data are presented as means ± standard error of the mean (SEM), unless otherwise specified.

## 5. Conclusions

Taken together, our postmortem and in vitro data support a model in which lactate accumulation and reduced brain pH act upstream of both *TGFB2* induction and a broader pH-sensitive transcriptional and epigenetic programing in iPSC-derived astrocytes and brainoids. Within this framework, *TGFB2* is best viewed as an amplifying node within astrocyte-enriched gene networks relevant to SCZ and BD, rather than as the sole upstream driver. The cell-type selectivity of the response—predominantly observed in astrocytes and organoids and largely absent in neurons—together with the reversibility of *TGFB2* induction by pH normalization, identifies astrocytic lactate/pH-buffering capacity as a candidate point of intervention. Coupled with converging evidence of elevated brain lactate in SCZ, BD, and other neuropsychiatric disorders, this lactate/pH–epigenetic axis represents a plausible mechanistic and therapeutic framework that should now be tested in adequately replicated organoid systems and in patient-derived iPSC models, with particular attention to the relative contributions of pH-dependent and lactate-signaling/lactylation-dependent mechanisms.

Histone lactylation is a recently characterized post-translational histone modification that is generally associated with transcriptional activation [47,48]. Its emergence as a metabolically sensitive epigenetic mark provides a plausible framework for understanding how shifts in cellular metabolism, such as increased glycolysis and lactate accumulation, can directly influence chromatin dynamics and transcriptional programs in astrocytes.

Accordingly, further mechanistic studies are warranted to directly test this hypothesis, including delineation of the genomic loci affected by histone lactylation and its interaction with other epigenetic modifications (e.g., DNA methylation and histone acetylation). Such investigations may provide critical insights into disease-relevant regulatory networks and facilitate the development of targeted therapeutic strategies aimed at modulating lactate signaling or epigenetic remodeling.

Moreover, given that elevated lactate levels are a hallmark of mitochondrial dysfunction and considering accumulating evidence implicating mitochondrial abnormalities in SCZ and other psychotic disorders (e.g., Roberts et al. [49]), future studies should also aim to elucidate the upstream mechanisms driving lactate accumulation and reduced brain pH. Understanding the directionality between mitochondrial impairment, metabolic reprogramming, and epigenetic dysregulation will be essential for constructing a comprehensive model of disease pathophysiology.

## Figures and Tables

**Table 1:** Gene expression changes in brainoids upon lactic acid treatment.

Gene	Fold Change	(*p* Value) SD
*TGFB1*	1.15	(0.09) 0.1
*TGFB2*	2.1	(**0.012**) 0.18
*CCND1*	1.3	(**0.01**) 0.04
*GALC*	1.15	(0.5) 0.23
*EN2*	1.35	(0.11) 0.16
*BDNF*	0.9	(0.7) 0.3
*NURR1 (NR4A2)*	0.2	(**0.008**) 0.1
*RELN*	1.7	(**0.04**) 0.18
*MAP2*	1.2	(**0.04**) 0.02
*GAD1*	1.43	(**0.004**) 0.04
*MKI67*	1.1	(0.15) 0.06
*CXCR4*	2.3	(**0.04**) 0.29
*OCT4 (POU5F1)*	0.44	(**0.025**) 0.13
*SNCA*	1.2	(0.5) 0.3
*DBH*	1.15	(0.5) 0.23
*MAOA*	1.35	(**0.01**) 0.05
*TPH2*	2.35	(**0.0005**) 0.04
*CDH2*	1.8	(**0.04**) 0.25
*HTR2A*	2.9	(**0.004**) 0.18
*DTNBP1*	0.45	(**0.04**) 0.14
*TH*	1.5	(**0.02**) 0.1
*SYN1*	1.34	(**0.004**) 0.02
*TNFA*	1.5	(**0.05**) 0.16
*NLRP3*	0.18	(**0.013**) 0.085
*IL13RA2*	2	(0.13) 0.6
*IFI16*	1.24	(0.25) 0.22
*SLC1A2*	1.7	(**0.025**) 0.16
*SLC16A1*	3.7	(**0.0005**) 0.1
*SLC16A7*	1.75	(**0.03**) 0.16
*DNMT1*	1.1	(0.2) 0.19
*DNMT3A*	1.3	(**0.05**) 0.08
*DNMT3B*	0.94	(0.4) 0.04
*SIRT1*	1.18	(0.3) 0.18
*IDH1*	1.25	(**0.045**) 0.07
*IDH2*	1.1	(**0.04**) 0.01

**Table 2:** DNA methylation changes as the result of lactic acid treatment in brainoids. Expression changes in brainoids and postmortem brain samples, as well as their relationships to pH and antipsychotic drug use, are presented in the two right columns for comparison.

Gene	5-mC Fold Change (p) SD	5-hmC Fold Change (p) SD [5-hmC% in Cont. vs. Lactic Acid]	Expression Fold Change (*p* Value) SD	Expression (Exp.) Status in Brain Tissues and Relation to pH and Antipsychotic Drug Use (If Present)
*TGFB1*	largely unmethylated	No 5-hmC	1.15 (0.09) 0.1	No Exp. change
*TGFB2*, site A	0.71 (**0.03**) 0.07	0.57 (**0.05**) 0.06 [2% vs. 1%]	2.15 (0.012) 0.4	Increased in SCZ and psychotic BD and low pH
*TGFB2*, site B	0.31 (**0.008**) 0.06	6 (**0.0001**) 0.05 [3% vs. 18%]		
*CCND1*	0.5 (**0.042**) 0.11	No 5-hmC	1.3 (0.01) 0.04	Increased at lower pH in controls, r = −0.46. Reduced expression in SCZ
*SLC16A1*	0.5 (**0.03**) 0.1	8.5 (**0.005**) 0.71 [0.8% vs. 7%]	3.6 (0.0004) 0.1	No change
*SLC16A7*	0.55 (**0.025**) 0.1	Negligible 5-hmC	1.75 (0.08) 0.16	Decreased in SCZ (*p* = 0.04)
*SLC1A2*	0.87 (NS)	7.4 (**0.00006**) 0.1 [0.3% vs. 2.1%]	1.7 (0.025) 0.16	Increased in SCZ and BD and at lower pH
*SLC1A3*	0.3 (**0.02**) 0.06	6.25 (**0.007**) 0.6 [0.8% vs. 5.4%]	1.3 10/28/25	Increased in SCZ and BD and low pH
*MAP2*	1.18 (NS)	No change	1.2 (0.04) 0.02	No Exp. change
*RELN*	1.3 (NS) 0.68	7.3 (**0.005**) 0.62 [0.8% vs. 6%]	1.7 (0.04) 0.18	No Exp. change in low pH
*NURR1*	2.3 (**0.045**) 0.3	No change	0.2 (0.007) 0.18	Trended decrease in SCZ, 30%
*EN2*	0.3 (**0.02**) 0.06	17 (0.004) 1.4 [0.2% vs. 3%]	1.34 (ns) 0.16	No Exp. change, but increased at low pH in controls, r = −0.55
*IFI16*	0.7 (**0.05**) 0.06	3.7 (**0.04**) 0.5 [0.3% vs. 1.5%]	1.25 (0.25) 0.22	No Exp. change.
*TNFA*	0.33 (**0.01**) 0.05	2.2 (**0.03**) 0.31 [1% vs. 2.2%]	1.5 (0.05) 0.02	No Exp. change, but increased at low pH (r = −0.5 and controls r = −0.65)
*MAOA*	0.58 (**0.012**) 0.11	1.85 (**0.025**) 0.19 [19% vs. 35%]	1.35 (0.067) 0.05	Increased in SCZ and BD and low pH. Antipsychotic drugs increase its Exp.
*IL6*	0.44 (0.12) 0.23	No 5-hmC	No expression	No Exp. change, but increases at low pH in controls, r = −0.49.
*CXCR4*	0.54 (**0.04**) 0.13	1.65 **(0.018)** 0.13 [1.5% vs. 2.4%]	2.3 (0.04) 0.29	No Exp. change, but correlates with pH in controls (*p* = 0.61) and inversely with antipsychotic drugs use (r = −0.37)
*NTRK2*	1.36 (NS)	No 5-hmC	Not done	Increased in SCZ and BD (>55%) and low pH (r = −0.42, in general) and correlate with *TGFB2* (r = 0.74)
*AUTS2*	3 (**0.01**) 0.17	3.5 (**0.03**) 0.56 [3.3% vs. 12%]	Not done	Increased in BD (19%, *p* = 0.03) and by antipsychotic drugs in BD (r = 0.88). Inverse correlation with *TGFB2* in BD (r = −0.55)
*MECP2*	1.95 (**0.05**) 0.31	Negligible	Not done	Its expression correlates with pH in controls (r = 0.65)
*TINF2*	1.8 (**0.027**) 0.09	16 (**0.0002**) 0.1 [2% vs. 31%]	Not done	No Exp. change and no correlation
*GRID2*	1.53 (0.07) 0.15	Negligible	Not done	No Exp. change and no correlation
*MBD4*	2.3 (**0.01**) 0.13	6 (**0.03**) 0.35 [1% vs. 6%]	Not done	No Exp. change, but correlation with pH in controls (r = 0.72)

## Data Availability

The data that support the findings of this study are available from the corresponding author upon reasonable request.

## Supplementary Materials

The following supporting information can be downloaded at https://www.mdpi.com/article/10.3390/ijms27125456/s1.

## Abbreviations

The following abbreviations are used in this manuscript.

AUTS2	autism susceptibility candidate 2
BD	bipolar disorder
BDNF	brain-derived neurotrophic factor
bFGF	basic fibroblast growth factor
BMP7	bone morphogenetic protein 7
BMPR1A	bone morphogenetic protein receptor type 1A
BMPR1B	bone morphogenetic protein receptor type 1B
CA1	carbonic anhydrase 1
CA2	carbonic anhydrase 2
CA3	carbonic anhydrase 3
CA9	carbonic anhydrase 9
CA12	carbonic anhydrase 12
CA13	carbonic anhydrase 13
CA14	carbonic anhydrase 14
CA5A	carbonic anhydrase 5A
CBP	CREB-binding protein
CCND1	cyclin D1
CDH2	cadherin 2
CREB	cAMP response element-binding protein
CXCR4	chemokine (C-X-C motif) receptor 4
DBH	dopamine β-hydroxylase
DMEM/F-12	Dulbecco’s modified Eagle medium F12
DNMTs	DNA (cytosine 5) methyltransferases
DNMT3A	DNA (cytosine 5) methyltransferase 3A
DNMT3B	DNA methyltransferase 3 beta
DNMT3L	DNA (cytosine 5) methyltransferase 3-like
DTNBP1	Dystrobrevin-binding protein 1
EGF	epidermal growth factor
EN-2	engrailed 2
EP300	histone acetyltransferase p300
FBS	fetal bovine serum
GAD1	glutamate decarboxylase 1
GALC	galactosylceramidase
GFAP	glial fibrillary acidic protein
GRID2	glutamate ionotropic receptor delta-type subunit 2
GSEA	gene set enrichment analysis
hEBs	human embryoid bodies
HCO_3_^−^	bicarbonate ion
HDACs	histone deacetylases
HIF1A	hypoxia-inducible factor 1 alpha
hiPSCs	human induced pluripotent stem cells
HTR2A	5-hydroxytryptamine receptor 2A
IDH1	isocitrate dehydrogenase 1
IDH2	isocitrate dehydrogenase 2
IFI16	gamma interferon-inducible protein ifi-16
IL6	interleukin 6
IL13RA2	interleukin 13 receptor subunit alpha 2
IPA	ingenuity pathway analysis
iPSCs	induced pluripotent stem cells
KDM3A	lysine (K)-specific demethylase 3A
KDM5B	lysine (K)-specific demethylase 5B
LDH	lactate dehydrogenase
LDHA	lactate dehydrogenase A
LDHB	lactate dehydrogenase B
LDHD	lactate dehydrogenase D
MAOA	monoamine oxidase A
MAP2	microtubule associated protein 2
MBD4	methyl CpG-binding domain protein 4
MECP2	methyl CpG-binding protein 2
METTL7A	methyltransferase-like 7A
MKI67	antigen Kiel 67
NaOH	sodium hydroxide
NLRP3	NLR family pyrin domain containing 3
NSCs	neural stem cells
NTRK2	neurotrophic receptor tyrosine kinase 2
NURR1	nuclear receptor-related 1 protein
OCT4	octamer-binding transcription factor 4
PMI	postmortem interval
qMSP	quantitative methylation-specific PCR
qPCR	quantitative PCR
qRT-PCR	quantitative real-time PCR
RELN	reelin
ROCK	rho kinase
SCFAs	short-chain fatty acids
SCZ	schizophrenia
SIRT1–7	sirtuin 1–7
SLC1A2	solute carrier family 1 member 2
SLC1A3	solute carrier family 1 member 3
SLC16A1	solute carrier family 16 member 1
SLC16A3	solute carrier family 16 member 3
SLC16A7	solute carrier family 16 member 7
SLC4A4	solute carrier family 4 member 4
SNCA	alpha-synuclein gene
SYN1	synapsin 1
TET	ten-eleven translocation
TGFβ	transforming growth factor beta
TGF-β2	transforming growth factor beta 2
TGFBR3	transforming growth factor beta receptor 3
TH	tyrosine hydroxylase
TINF2	TERF1-interacting nuclear factor 2
TNF-alpha	tumor necrosis factor alpha
TPH2	tryptophan hydroxylase isoform 2
T4-BGT	T4 phage β-glucosyltransferase
