# Supplementary material for: Lactate-Mediated Brain Acidosis Drives Epigenetic Dysregulation of TGFB2 and Associated Gene Networks in Schizophrenia and Bipolar Disorder
Source: Int J Mol Sci. 2026 Jun 17;27(12):5456. doi: 10.3390/ijms27125456 (PMC13299459; doi:10.3390/ijms27125456)
Supplement: Supplementary file 1 [file ijms-27-05456-s001.zip › Supplementary Figures S1.pdf]

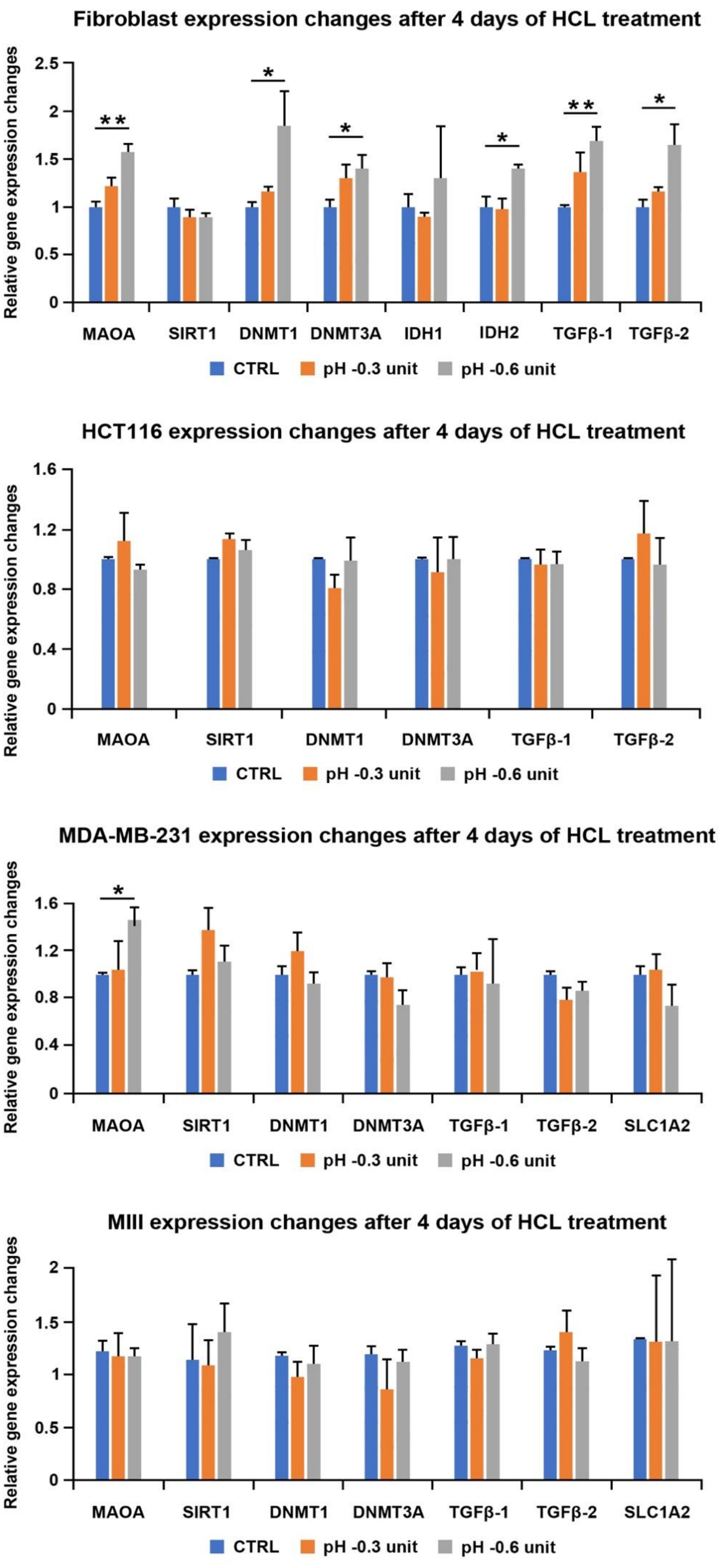

Supplementary Figure S1. A. Expression changes of key genes in fibroblasts following four days of HCl treatment, resulting in a 0.3 and 0.6 unit decrease in culture medium pH. B–D. The same experimental design applied to cancer cell lines. Fibroblasts showed increased gene expression, particularly under the higher HCl dose, whereas this trend was largely absent in all three cancer cell lines.
