# Supplementary material for: Lactate-Mediated Brain Acidosis Drives Epigenetic Dysregulation of TGFB2 and Associated Gene Networks in Schizophrenia and Bipolar Disorder
Source: Int J Mol Sci. 2026 Jun 17;27(12):5456. doi: 10.3390/ijms27125456 (PMC13299459; doi:10.3390/ijms27125456)
Supplement: Supplementary file 1 [file ijms-27-05456-s001.zip › Supplementary Tables S1 and S2.pdf]

Supplementary Table S1. Primers used for gene expression analysis

Gene	Forward primer	Reverse primer	Amplicon size (bp)
**β-Actin**	CGAGCACAGAGCCTCGCCTTTGCC	TGTCGACGACGAGCGCGGCGATAT	94
**BDNF**	CTACGAGACCAAGTGCAATCC	AATCGCCAGCCAATTCTCTCTT	147
**CDH2**	AGCCAACCTTAACTGAGGAGT	GGCAAGTTGATTGGAGGGATG	136
**CCND1**	GCTGCGAAGTGGAAACCATC	CCTCCTTCTGCACACATTTGAA	135
**CXCR4**	ACTACACCGAGGAAATGGGCT	CCCACAATGCCAGTTAAGAAGA	133
**DBH**	TCGTGGTGCTCTGGACCGATGG	GGATCCAGGTGGATCTGCCCCTT	81
**DNMT1**	CCTAGCCCCAGGATTACAAGG	ACTCATCCGATTTGGCTCTTTC	118
**DNMT3A**	CCGATGCTGGGGACAAGAAT	CCCGTCATCCACCAAGACAC	151
**DNMT3B**	ACCTCGTGTGGGGAAAGATCA	CCATCGCCAAACCACTGGA	121
**DTNBP1**	TGGTGGATAGCGAGGTGGTC	CTCCTCAAAACTCGCCTCTAAATGA	159
**EN2**	CCGGCGTGGGTCTACTGTA	CCTCTTTGTTCGGGTTCTTCTT	91
**GALC**	TATTTCCGAGGATACGAGTGGT	CCAGTCGAAACCTTTTCCCAG	111
**GAD1**	GCGGACCCCAATACCACTAAC	CACAAGGCGACTCTTCTCTTC	144
**GAPDH**	ACAACTTTGGTATCGTGGAAGG	GCCATCACGCCACAGTTTC	101
**GFAP**	AGGTCCATGTGGAGCTTGAC	GCCATTGCCTCATACTGCGT	82
**GRID2**	TGATGAGGTATTTCGCACTGC	GGCCAAGATGCCTTGATTCATAA	157
**HIF1A**	GAACGTCGAAAAGAAAAGTCTCG	CCTTATCAAGATGCGAACTCACA	124
**HTR2A**	TCATGCCCGTGTCCATGTTAACCAT	GAGCACGTCCAGGTAAATCCAGACT	100
**IDH1**	TGTGGTAGAGATGCAAGGAGA	TTGGTGACTTGGTCGTTGGTG	147
**IDH2**	CCCGTATTATCTGGCAGTTCATC	TCCACATCTTCTTCAGCTTGAAC	104
**IFI16**	TAGAAGTGCCAGCGTAACTCC	TGATTGTGGTCAGTCGTCCAT	179
**IL13RA2**	ACCTGGCATAGGTGTACTTCT	CCAAATAGGGAAATCTGCATCCT	134
**IL6**	ACTCACCTCTTCAGAACGAATTG	CCATCTTTGGAAGGTTCAGGTTG	149
**MAOA**	TGTTCGACGTAGTCGTGATCGGAGG	GTTCTTCCTCCAACCCTGTCCCGAG	151
**MAP2**	CGAAGCGCCAATGGATTCC	TGAACTATCCTTGCAGACACCT	161
**MKI67**	GCCTGCTCGACCCTACAGA	GCTTGTCAACTGCGGTTGC	127
**NLRP3**	CCACAAGATCGTGAGAAAACCC	CGGTCCTATGTGCTCGTCA	921
**NTRK2**	ACCCGAAACAAACTGACGAGT	AGCATGTAAATGGATTGCCCA	91
**NURR1 (NR4A2)**	GCACTCCGGGTCGGTTTAC	GCCACGTAGTTCTGGTGGAA	129
**RELN**	GCGAGGTGCTCATTTCCCTGCATATT	GTGTATAGTCCTGTCACCAGCAAGC	124
**OCT4**	GGGAGATTGATAACTGGTGTGTT	GTGTATATCCCAGGGTGATCCTC	144
**SIRT1**	TGCGGGAATCCAAAGGATAATTCAGTGTC	CTTCATCTTTGTCATACTTCATGGCTCTATG	236
**SLC16A1**	AGTAGTTATGGGAAGAGTCAGCA	GTCGGGCTACCATGTCAACA	88
**SLC16A7**	TAGCAGGAGGCTTATTATGCTGT	GGTTGAAGGCTAAACCTAAAC	114
**SLC1A2**	CCTTGTCCAAGCCTGCTTTCA	CTCAGTCACAGTCTCGTTCAAC	127
**SLC1A3**	AGCAGGGAGTCCGTAAACG	AGCATTCCGAAACAGGTAACTTT	91
**SNCA**	GACAAAAGAGGGTGTTCTCTATGTAG	GCTCCTCCAACATTTGTCACTT	111
**SYN1**	AGTTCTTCGGAATGGGGTGAA	CAAACTGCGGTAGTCTCCGTT	106
**TGFB1**	CTAATGGTGGAAACCCACAACG	TATCGCCAGGAATTGTTGCTG	209
**TGFB2**	AGAGTGCCTGAACAACGGATT	CCATTCGCCTTCTGCTCTT	116
**TH**	GGGCTGTGTAAGCAGAACG	AAGGCCCGAATCTCAGGCT	107
**TPH2**	ACGGAGAGGGTTTTCCCTG	GCCTTTGTCGTCATTTTTGCCA	103
**TNFA**	GAGGCCAAGCCCTGGTATG	CGGGCCGATTGATCTCAGC	91

Note: The sequence of primers taken from the Harvard primer bank with the exception of β-actin, BIRC5, TGFB2, MAOA, SIRT1 and RELN

Supplementary Table S2. Primers used for 5-mc and 5-hmc analysis designed from the promoter regions of the candidate genes			
**Gene**	**Forward primer**	**Reverse primer**	**Amplicon size (bp)**
**AUTS2**	AGTTTCGCCCTCTCTTCCGCTAATGA	AAACTCACTCCCAGGCTCCCAAAT	148
**CXCR4**	CACCTGTCTTCAGGCGCATC	GGACCCTGCTGTTTGCGGGT	106
**EN2**	AACGGGGTTCCCGGGTCAGT	GAACGACCGCCGCCCTCAAG	109
**GRID2**	AAGAAAGTGTTGGTGCAGCTCGTG	ACTGAAGCCACCTCCAACAAGACA	112
**IL6**	AGGTGGGTAGGCTTGGC	CGTTGGCCTCAAATCTACAGG	134
**IFI16**	CTCCTATTATAAAGTTTGCTTTTTTGGC	ACAGGCACACATGACCATAC	187
**MAOA**	GACCTCGACGGGCCCTACA	AGCCAGGCAGGATCGCAGGGTCG	93
**MBD4**	CTTTGCCGCTCTCCAACCCTC	AAGAGCAGGAGGTCGAGTCCT	135
**MECP2**	AAATGGACAGGAAATCTCGCCAAT	CCGTCATTGGCTGTGATG	99
**NTRK2**	GCTCTCTCGAGAGAGAAGGATTG	ACACTTCCGAGGGCTCTGGTC	238
**NURR1**	CATCCCAGGGTGCGGGAA	GCAGCCTCCCAAGTCTCC	190
**RELN**	TTTGACGTCCCTCGCAGAAGAGT	TGGCTCGGCGGCACCT	165
**SLC1A2**	CGTTGAGGCGCTAAAGGGCTT	CTCACCCTTCCGTAGATGCCAT	186
**SLC1A3**	GTTAAATCAACTTTAAGAGGAAGAGG	GCTGGTCTTCAAACACCCAAC	113
**SLC16A1**	TCGGTTCTACTACTGTCGCCAC	ATTTGAATGGATGCGGGTGCC	165
**SLC16A7**	CTTTGACAGCCGCTTGGAGCA	CTCGTGAACGCCCTTGTGG	149
**TGFB1**	TTCCATCCTTCAGGTGTCCTGTTG	GGTGTGGGTCACCAGAGAAAGAG	205
**TGFB2, site A**	CCTTTTACCATGAAGACTGTAGAGAC	TCAAATGATCAGTTCTTTGAAGACCT	170
**TGFB2, site B**	CGTGGTTCAGAGAGAACTTATAAATCTCC	TCTGTCTTTCTCTTGTCAGGAGC	119
**TINF2**	CTCAGCCGCCAACTCATGTCAG	CCGAGGGTGCCTCACTTC	122
**TNFA**	AAGACTGAAACCAGCATTATGAG	GGACAAGCCTGGGACAGC	118
EN2 primers sequences taken from James et al., 2014			
